# Supplementary material for: Saposin Lipid Nanoparticles: A Highly Versatile and Modular Tool for Membrane Protein Research
Source: Structure. 2018 Feb 6;26(2):345–355.e5. doi: 10.1016/j.str.2018.01.007 (PMC5807053; doi:10.1016/j.str.2018.01.007)
Supplement: Document S1. Figures S1–S5 and Tables S1–S4 [file mmc1.pdf]

Structure, Volume *26*

Supplemental Information

Saposin Lipid Nanoparticles: A Highly Versatile and Modular Tool for Membrane Protein Research

Ali Flayhan, Haydyn D.T. Mertens, Yonca Ural-Blimke, Maria Martinez Molledo, Dmitri I. Svergun, and Christian Löw

Saposin lipid nanoparticles: a highly versatile and modular tool for membrane protein research

Ali Flayhan[1], Haydyn D. T. Mertens[2], Yonca Ural-Blimke[1], Maria Martinez Molledo[1], Dmitri I. Svergun[2] and Christian Löw*[1, 3, 4].

[1] Centre for Structural Systems Biology (CSSB), DESY and European Molecular Biology Laboratory Hamburg, Notkestrasse 85, D-22607 Hamburg, Germany.

[2] European Molecular Biology Laboratory Hamburg, Notkestrasse 85, D-22607 Hamburg, Germany.

[3] Department of Medical Biochemistry and Biophysics, Karolinska Institutet, Scheeles väg 2, SE-17177 Stockholm, Sweden.

[4]Lead contact

* Correspondence: christian.loew@embl-hamburg.de

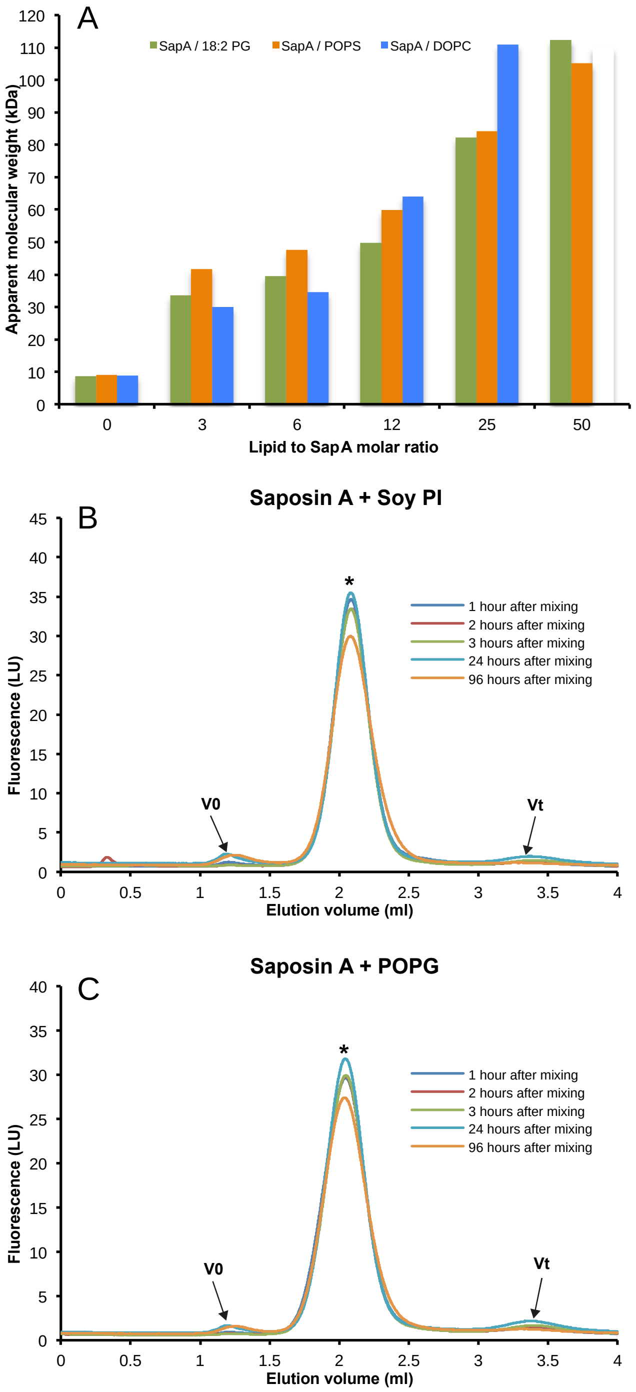

Figure S1. Related to Figure 1. (A) Positive correlation between the molecular weight of the nanoparticles and the lipid to saposin molar ratio. The lipid to SapA molar ratios are indicated on the X-axis and their corresponding apparent molecular weights, estimated by size-exclusion chromatography, on the Y-axis. The different SapA/lipid systems are color-coded as indicated in the figure. (B - C) Stability of SapNPs over time. Size-exclusion chromatography of SapA after incubation with detergent-solubilized SoyPI (B) and POPG (C), respectively. SapNPs (*) have been injected on the gel filtration column after different time points as indicated in the figure. The black arrows represent the void volume (V0) and the total volume (Vt) of the column.

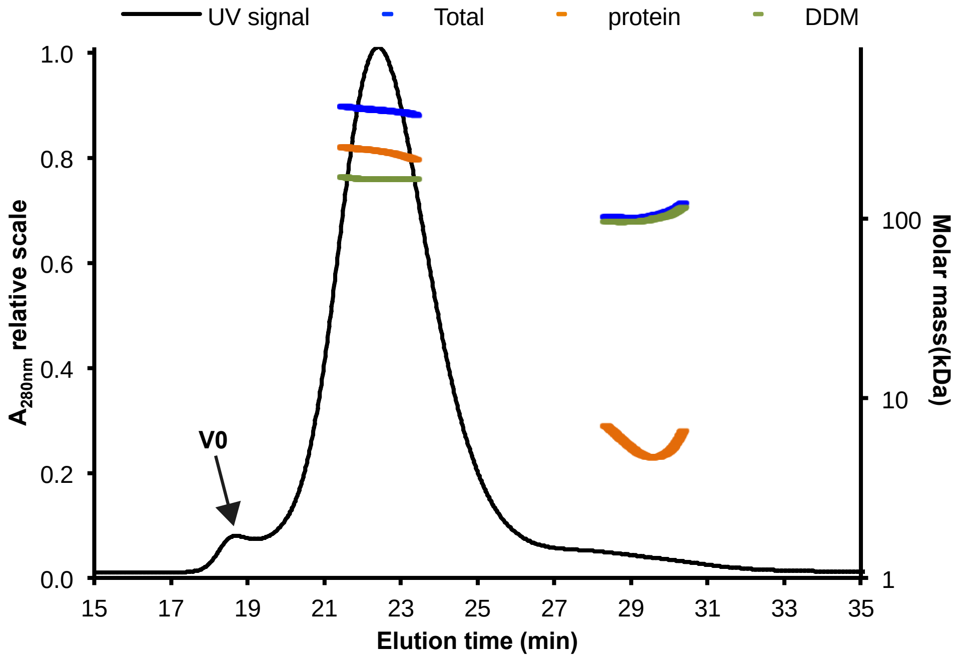

Figure S2. Related to Figure 3 and 6. Triple detection size-exclusion chromatography conjugate analysis of the T2 channel in DDM. The UV absorbance at 280 nm and the derived molar masses (logarithmic scale) of T2 (orange), DDM (green) and T2/DDM protein/detergent complex (blue) are plotted with respect to the elution time. The black arrow represents the void volume (V0) of the column. 60 µL of T2 at 5 mg/mL were injected onto a home-packed SD200 10/300 column pre-equilibrated with 20 mM Tris pH 7.5, 150 mM NaCl, 5 % glycerol, and 0.03 % DDM at a flow rate of 0.5 mL/min. Note that the second peak contains only the excess of DDM micelles.

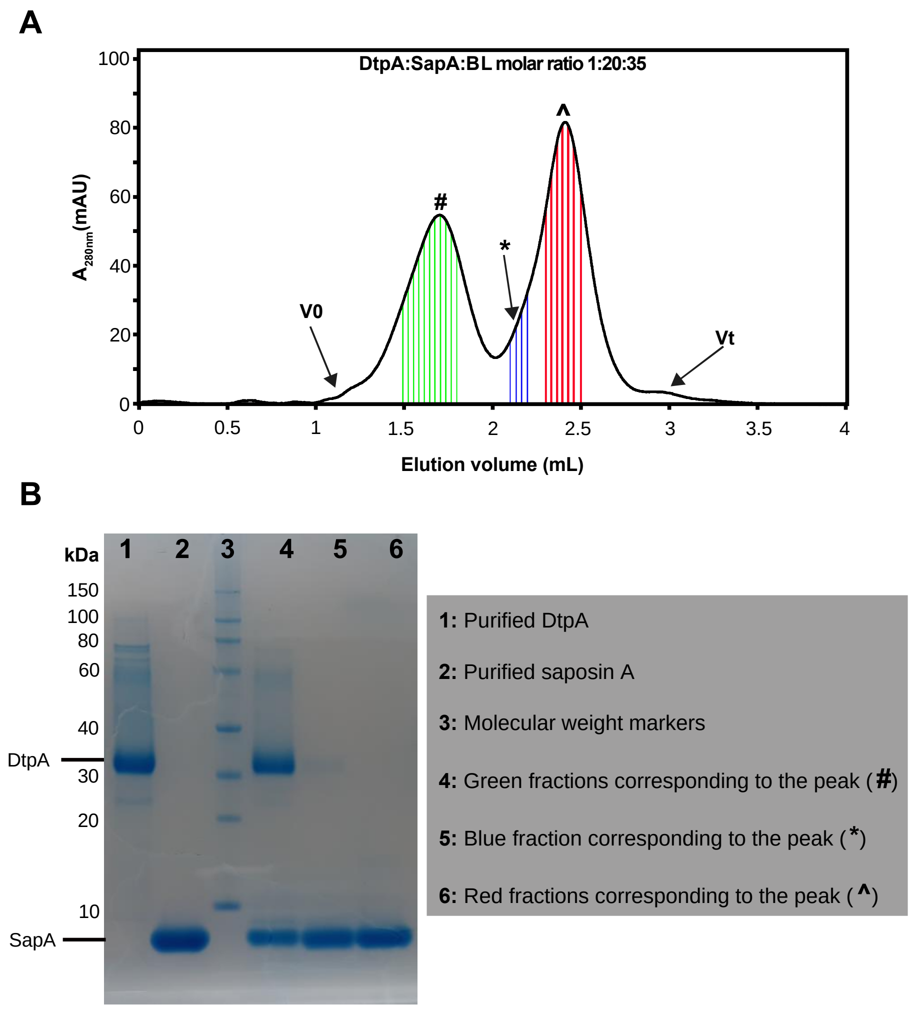

Figure S3. Related to Figure 3. (A) Gel filtration elution profile of DtpA reconstituted in SapA/BL nanopartilces. The DtpA:SapA:lipid molar ratio is 1:20:35. The free SapA peak is marked with (^), the empty nanoparticles with (*), and the reconstituted DtpA with (#). The black arrows represent the void (V0) and the total (Vt) volumes of the column used. (B) SDS-PAGE analysis of the chromatographic peaks. As seen in lanes 4 to 6, the first peak (green in panel A), assigned for the reconstituted protein, contains both DtpA and saposin A, the second and third peaks (blue and red in first panel), assigned for empty nanoparticles and free SapA, respectively, contain only SapA.

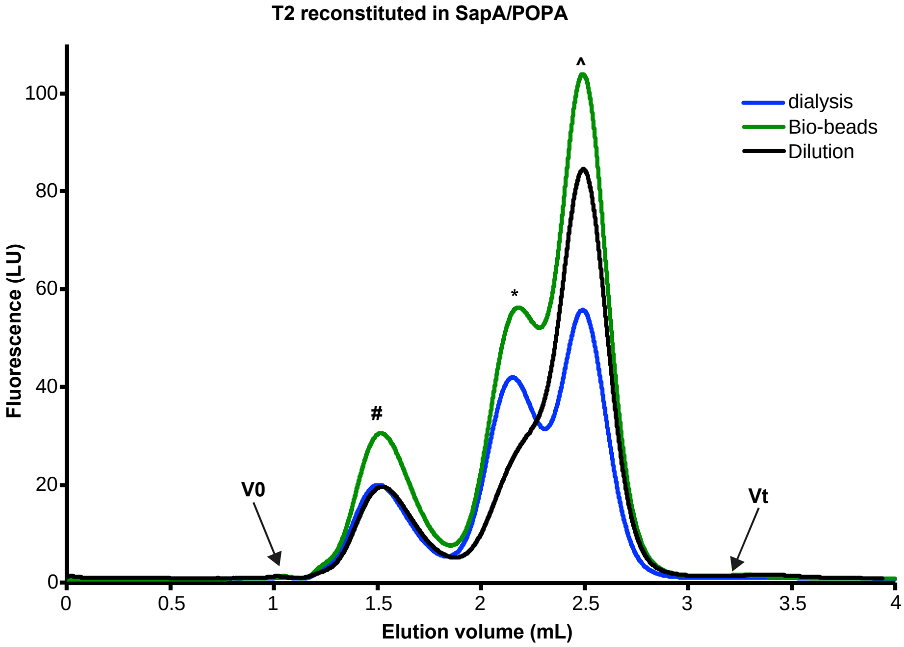

Figure S4. Related to Figure 3 and 4. Detergent removal after reconstitution. The gel filtration profiles of the mechanosensitive T2 channel after reconstitution in SapA/POPA are shown. DDM removal was carried out by dilution, dialysis, and bio-beads as indicated in the figure. The free SapA peak is marked with (^), the empty nanoparticles with (*), and the reconstituted T2 with (#). The black arrows represent the void volume (V0) and the total volume (Vt) of the column.

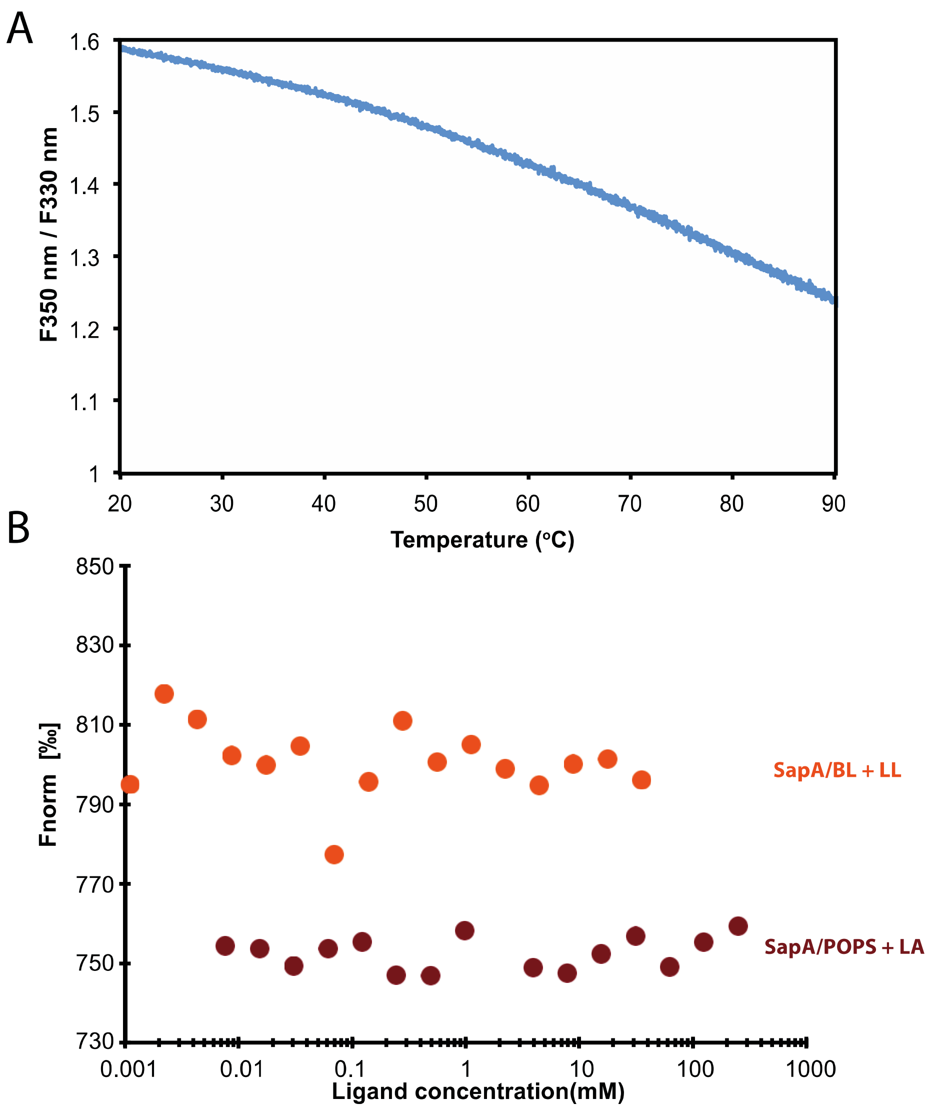

Figure S5. Related to Figure 4. (A) The experimental melting curve of purified SapA obtained with dye-free nanoDSF. Note that, the fluorescence signal of saposin A is temperature dependent as expected but does not show any obvious unfolding transition. (B) MST measurements of the binding of Leu-Leu and Leu-Ala dipeptides to SapA/BL and SapA/POPS empty nanoparticles, respectively. Unlike for the reconstituted proteins, no peptide binding could be observed with empty nanoparticles.

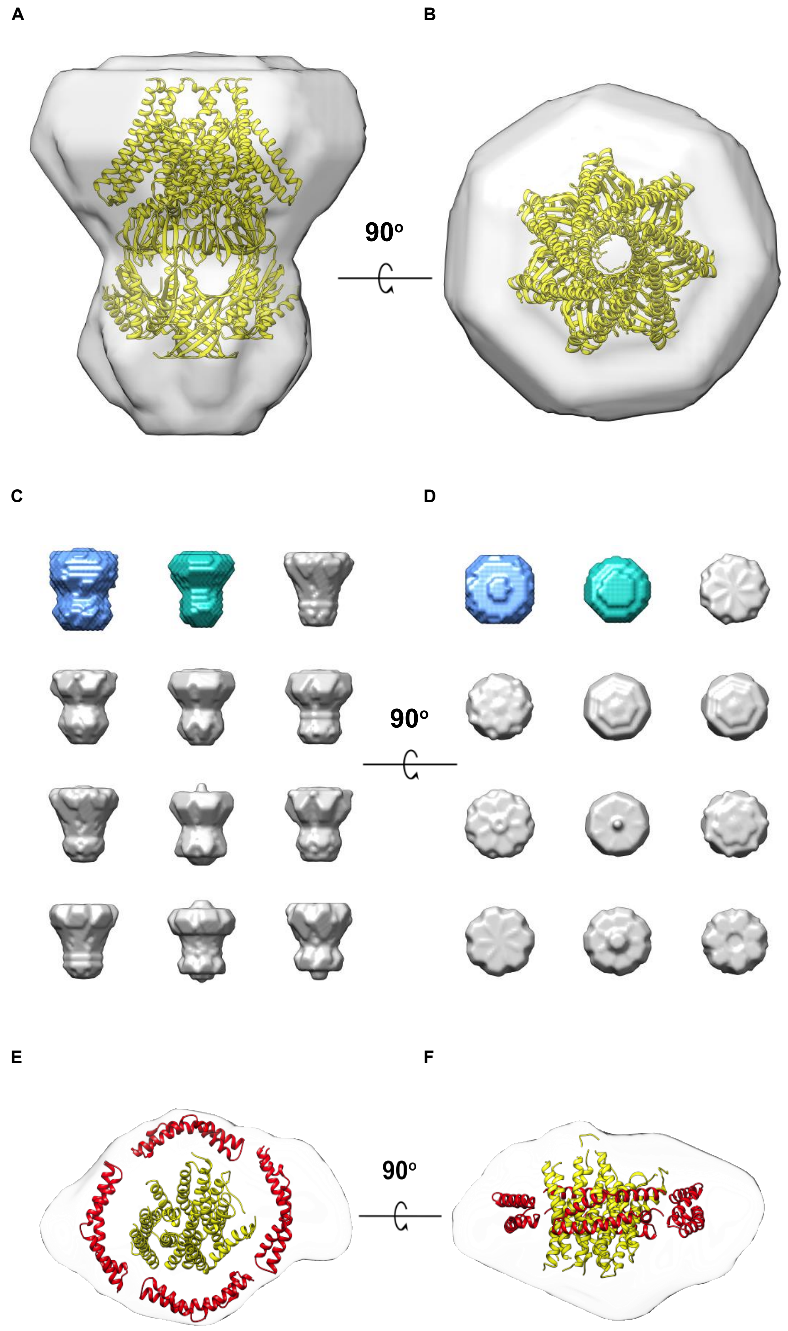

Figure S6. Related to Figure 6. (A – B) The *ab initio* model generated for the T2 channel in DDM with a homologous structure (PDB ID: 3T9N) fitted in the shape volume. Ten independent reconstructions were generated using DAMMIF (C and D, in gray) and the program DAMMAVER was used to generate the average representative models (C and D, average model blue and volume filtered cyan). *The* D_{max} of the particles is ~ 165 Å. (E – F) The best representative *ab initio* model generated for DtpA in SapA:POPS. Four SapA molecules (PDB ID: 4DDJ) and one homologous transporter molecule (PDB ID: 4Q65) were docked manually into the volume.

Table S1: A list of the lipids used for SapNPs formation. **Related to the STAR Methods section.**

Lipid name	Abbreviation	Percentage of DDM (w/v) used to solubilize the lipid at 5 mg/ml
L-α-phosphatidylinositol (Soy)	Soy-PI	0.5
1-palmitoyl-2-oleoyl-*sn*-glycero-3-phospho-L-serine	POPS	0.8
1-palmitoyl-2-oleoyl-*sn*-glycero-3-phospho-(1'-*rac*-glycerol)	POPG	0.6
1-palmitoyl-2-oleoyl-*sn*-glycero-3-phosphoethanolamine	POPE	1
1-palmitoyl-2-oleoyl-*sn*-glycero-3-phosphate	POPA	1
1,2-Dioleoyl-sn-glycero-3-phosphocholine	DOPC	1
1,2-dilinoleoyl-*sn*-glycero-3-phospho-(1'-*rac*-glycerol)	18:2 PG	0.8
1,2-dilinoleoyl-*sn*-glycero-3-phosphoethanolamine	18:2 PE	2.2
1,2-dilinoleoyl-*sn*-glycero-3-phosphate	18:2 PA	1.2
1,2-dilinoleoyl-*sn*-glycero-3-phosphocholine	18:2 (cis) PC	1.2
1-myristoyl-2-palmitoyl-*sn*-glycero-3-phosphocholine	MPPC	0.6
1,2-dibehenoyl-*sn*-glycero-3-phosphocholine	22:0 PC	n/a
1,2-dilauroyl-*sn*-glycero-3-phospho-(1'-*rac*-glycerol)	DLPG	0.6
D-lactosyl-ß-1,1'-N-stearoyl-D-*erythro*-sphingosine	C18 ß-D-Lactosyl Ceramide	2 and 2.5 % LDAO
D-galactosyl-ß-1,1'-N-stearoyl-D-*erythro*-sphingosine	C18 ß-D-Galactosyl Ceramide	2 and 2.5 % LDAO
1',3'-bis[1,2-dioleoyl-*sn*-glycero-3-phospho]-*sn*-glycerol	18:1 Cardiolipin	2.5 and 0.5 % LDAO
E. coli polar lipid extract	n/a	0.7
Brain total lipid extract (porcine)	BL	0.28

Table S2: Screening of different molar ratios to reconstitute membrane proteins into SapNPs**. Related to Figure 3.** Abbreviations: MP: membrane protein.

MP	Lipid	Saposin	Molar ratios	
			**Lipid to MP**	**Saposin to MP**
T2	Soy PI	SapA	40	10
				15
				20
				25
T2	POPA	SapA	40	10
				15
				20
				25
T2	POPS	SapA	40	10
				15
				20
				25
T2	DOPC	SapA	40	10
				15
				20
				25
$PepT_{SO2}$ and $PepT_{St}$	BL	SapA	35	10
				15
			70	10
				15
$PepT_{SO2}$ and $PepT_{St}$	POPA	SapA	35	10
				15
			70	10
				15
$PepT_{SO2}$ and $PepT_{St}$	POPS	SapA	35	10
				15
			70	10
				15
DtpA	BL	SapA	35	10
				15
				20
			70	10
				15
				20
DtpA	POPA	SapA	35	10
				15
				20
			70	10
				15
				20
DtpA	POPS	SapA	35	10
				15
				20
			70	10
				15
				20

Table S3: SAXS data collection and derived parameters for empty SapNPs. **Related to Figure 2.** Abbreviations: R_g: radius of gyration; D_{max}: maximal particle dimension.

	**SapA/DOPC**	**SapA/SoyPI**	**SapA/POPG**
**Data collection parameters**			
Instrument	EMBL P12 beam line (PETRA-III, DESY, Hamburg)		
Beam geometry	0.2 x 0.12 mm^2		
Wavelength (Å)	1.24		
s range ($Å^{-1}$)[a]	0.01-0.46		
Collection mode	Batch	SEC-SAXS	
Exposure time (s)	1 (20×0.05 s)	3600 (3600 x 1 s)	
OD_{280nm} (A.U.)	4.3	5.1	6.7
Temperature (K)	283		
**Structural parameters**			
I(0) (A.U.) (from $p(r)$)	39720 ± 51	42510 ± 37	823 ± 2
R_g (Å) (from $p(r)$)	39.9 ± 0.5	33.9 ± 0.2	40.4 ± 0.7
I(0) (A.U.) (from Guinier)	40271 ± 55	43388 ± 36	818 ± 3
R_g (Å) (from Guinier)	41.2± 0.1	35.9 ± 0.2	40.5 ± 0.2
D_{max} (Å)	115 ± 5	85 ± 5	105 ± 5
Porod volume estimate (x10^3 $Å^3$)	96 ± 10	68 ± 7	69 ± 7
Protein dry volume calculated from sequence ($Å^3$)[b]	11160		
**Software employed**			
Primary data reduction	RADAVER		
Data processing	PRIMUS/Qt		
Ab initio analysis	DAMMIF, DAMMIN		
Validation and averaging	DAMAVER		
Rigid-body modeling	CORAL		
Computation of model intensities	CRYSOL		
3D graphics representations	PyMOL, UCSF Chimera		

[a]Momentum transfer $s = 4\pi\sin(\theta)/\lambda$. [b]Dry volume determined using the server: http://www.molmovdb.org/cgi-bin/psv.cgi

Table S4: SAXS data collection and derived parameters for the T2 channel and DtpA in DDM or SapNPs. **Related to Figure 6.** Abbreviations: R_g: radius of gyration; D_{max}: maximal particle dimension.

	**T2 DDM**	**T2 SapA:POPS**	**DtpA SapA:POPS**
**Data collection parameters**			
Instrument	EMBL P12 beam line (PETRA-III, DESY, Hamburg)		
Beam geometry	0.2 x 0.12 mm^2		
Wavelength (Å)	1.24		
s range ($Å^{-1}$)[a]	0.01-0.46		
Collection mode	SEC-SAXS		
Exposure time (s)	3600 (3600 x 1 s)		
OD_{280nm} (A.U.)	10.4	3.6	11.8
Temperature (K)	283		
**Structural parameters**			
I(0) (A.U.) (from $p(r)$)	250 ± 1.0	2572 ± 5	3289 ± 3
R_g (Å) (from $p(r)$)	55.6 ± 0.2	53.9 ± 0.2	39.7 ± 0.4
I(0) (A.U.) (from Guinier)	251.22 ± 5	2563.72 ± 5	3312 ± 3
R_g (Å) (from Guinier)	56.5 ± 0.1	53.5 ± 0.2	40.2 ± 0.1
D_{max} (Å)	165 ± 5	180 ± 5	135 ± 5
Porod volume estimate (x10^3 $Å^3$)	896 ± 90	686 ± 70	288 ± 30
Protein dry volume calculated from sequence ($Å^3$)[b]	42042.5		65185.8
**Software employed**			
Primary data reduction	RADAVER		
Data processing	PRIMUS/Qt		
Ab initio analysis	DAMMIF, DAMMIN		
Validation and averaging	DAMAVER		
Rigid-body modeling	CORAL		
Computation of model intensities	CRYSOL		
3D graphics representations	PyMOL, UCSF Chimera		

[a]Momentum transfer $s = 4\pi\sin(\theta)/\lambda$. [b]Dry volume determined using the server: http://www.molmovdb.org/cgi-bin/psv.cgi
