# Supplementary material for: Saposin Lipid Nanoparticles: A Highly Versatile and Modular Tool for Membrane Protein Research
Source: Structure. 2018 Feb 6;26(2):345–355.e5. doi: 10.1016/j.str.2018.01.007 (PMC5807053; doi:10.1016/j.str.2018.01.007)
Supplement: Document S2. Article plus Supplemental Information [file mmc2.pdf]

Structure

Resource

Saposin Lipid Nanoparticles: A Highly Versatile and Modular Tool for Membrane Protein Research

Graphical Abstract

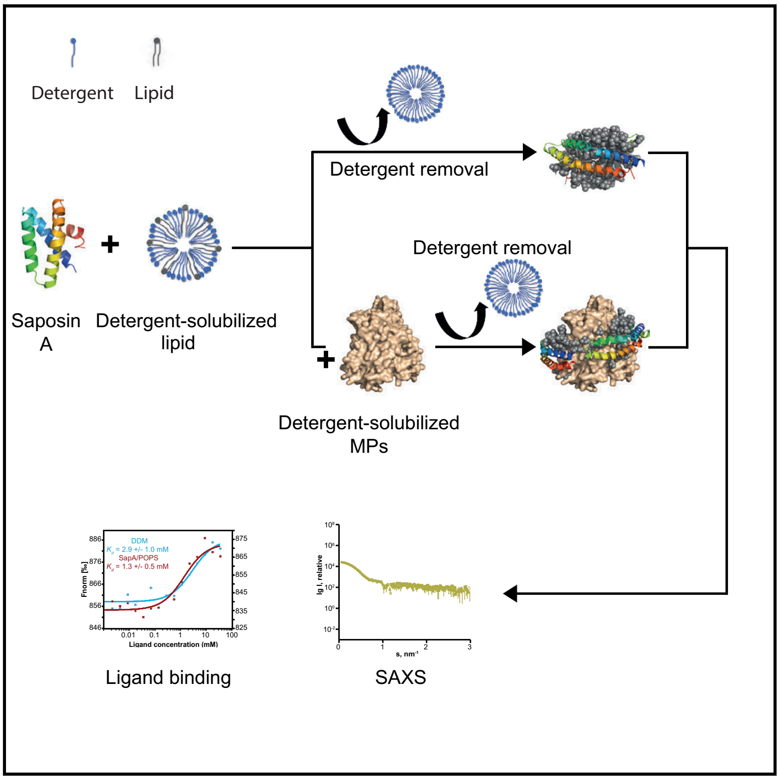

Highlights

- SapA shows the lowest lipid specificity for SapNP formation
- SapNPs are versatile and can adapt to MPs of various sizes and architectures
- SapNP-reconstituted MPs are more stable than in detergent
- SapNPs encapsulate MPs in a compact manner

Authors

Ali Flayhan, Haydyn D.T. Mertens, Yonca Ural-Blimke, Maria Martinez Molledo, Dmitri I. Svergun, Christian Löw

Correspondence

christian.loew@embl-hamburg.de

In Brief

Saposin-derived lipid nanoparticles (SapNPs) are an alternative tool for membrane protein (MP) reconstitution. Flayhan et al. characterize these nanoparticles, showing that they are versatile and can adapt to MPs of various sizes and architectures. SapNP-reconstituted MPs can be treated as soluble proteins and used for structural and functional studies.

Flayhan et al., 2018, Structure *26*, 345–355
February 6, 2018 © 2018 The Author(s). Published by Elsevier Ltd.
https://doi.org/10.1016/j.str.2018.01.007

Check for updates
CellPress

Structure
Resource

Saposin Lipid Nanoparticles: A Highly Versatile and Modular Tool for Membrane Protein Research

Ali Flayhan,[1,2] Haydyn D.T. Mertens,[2] Yonca Ural-Blimke,[1,2] Maria Martinez Molledo,[1,2] Dmitri I. Svergun,[2] and Christian Löw[1,2,3,4,*]
[1]Centre for Structural Systems Biology (CSSB), DESY, Notkestrasse 85, 22607 Hamburg, Germany
[2]European Molecular Biology Laboratory Hamburg, Notkestrasse 85, 22607 Hamburg, Germany
[3]Department of Medical Biochemistry and Biophysics, Karolinska Institutet, Scheeles väg 2, 17177 Stockholm, Sweden
[4]Lead Contact
*Correspondence: christian.loew@embl-hamburg.de
https://doi.org/10.1016/j.str.2018.01.007

SUMMARY

Saposin-derived lipid nanoparticles (SapNPs) are a new alternative tool for membrane protein reconstitution. Here we demonstrate the potential and advantages of SapNPs. We show that SapA has the lowest lipid specificity for SapNP formation. These nanoparticles are modular and offer a tunable range of size and composition depending on the stoichiometric ratio of lipid and saposin components. They are stable and exhibit features typical of lipid-bilayer systems. Our data suggest that SapNPs are versatile and can adapt to membrane proteins of various sizes and architectures. Using SapA and various types of lipids we could reconstitute membrane proteins of different transmembrane cross-sectional areas (from 14 to 56 transmembrane α helices). SapNP-reconstituted proteins bound their respective ligands and were more heat stable compared with the detergent-solubilized form. Moreover, SapNPs encircle membrane proteins in a compact way, allowing structural investigations of small membrane proteins in a detergent-free environment using small-angle X-ray scattering.

INTRODUCTION

Membrane proteins (MPs) are encoded by 20%–30% of open reading frames in sequenced genomes (Wallin and von Heijne, 1998) and provide key functions for inter- and intracellular communication. They play diverse and critical roles such as environmental change-induced signaling (Conde et al., 2011; Giuliani et al., 2011; Osakabe et al., 2013), nutrient and water uptake (Verkman and Mitra, 2000; Knütter et al., 2004; Guettou et al., 2013), mechanosensation (Haswell et al., 2011; Sukharev and Sachs, 2012), cell-to-cell adhesion, and communication (Lodish et al., 2000). MPs are also hijacked by pathogens and used as a first entry point into host cells (Grove and Marsh, 2011; Flayhan et al., 2012). Integral MPs are compartmentalized in natural lipid-bilayer membranes, which shield their hydrophobic transmembrane regions from the surrounding hydrophilic environment. Therefore, isolating and studying MPs in aqueous buffers are notoriously difficult tasks and require the use of substituting molecules to compensate for the nursing hydrophobic acyl chains of the lipids and to prevent aggregation. To date, this is mainly achieved by the use of detergents (Duquesne and Strugis, 2010), which, by definition, are amphipathic surfactants able to solubilize lipids. Nonetheless, due to their dissociating properties, detergents recurrently strip off structurally and functionally important lipids in a process called delipidation. In addition, detergents can hinder intraprotein interactions, resulting in MP instability and loss of function (Bowie, 2001; Garavito and Fergusson-Miller, 2001). In an attempt to overcome the hurdles associated with the use of detergent, various alternative surfactant molecules or polymers have been developed. Such tools include fluorinated surfactants (FSs) and amphipols (Breyton et al., 2010), both of which were shown to increase the biochemical and physical stability of MPs, compared with detergents (Kleinschmidt and Popot, 2014; Abla et al., 2015), and have been successfully used in structural studies including small-angle neutron scattering (Breyton et al., 2013a, 2013b) and single-particle electron microscopy (Liao et al., 2013; Beckham et al., 2017). Despite providing increased stability, these tools do have noticeable drawbacks. MP heterogeneity and aggregation have been associated with the use of FSs, which are difficult to synthesize and often not commercially available. The most commonly used amphipol, A8-35, can be used only at pH 7.0 or above and in the absence of multivalent cations, which otherwise will lead to the aggregation of the polymer itself and the MP/A8-35 complexes (Zoonens and Popot, 2014). Most importantly, these amphipathic molecules do not mimic all features of a lipid bilayer. The importance of lipids for MP stability and activity has been well established, but until recently the vital roles played by MP-associated lipids at the molecular level have not been studied in detail (Aryal et al., 2017; Norimastu et al., 2017). To understand MP structure and function in the context of a lipid bilayer it is essential to conduct biochemical, biophysical, and structural studies in a lipid environment. This can be done by extracting and isolating the MP of interest in styrene maleic acid copolymer lipid particles (SMALPs) (Lee et al., 2016), which provide the advantage of keeping the protein within a native lipid environment and avoiding complications arising from detergent exposure. A limitation of this method is that larger MPs or MP complexes cannot be extracted due to the SMALPs' maximal diameter size of around 15 nm. Moreover, the SMA

This is an open access article under the CC BY license (http://creativecommons.org/licenses/by/4.0/).

polymer has a limited solubility at pH values below 6.5–7 and at relatively high concentrations (>5 mM) of divalent ions (Lee et al., 2016). As an alternative approach, lipid particles, such as the apolipoprotein- and the membrane scaffold protein (MSP)-derived particles, also known as nanodiscs (NDs), have emerged as promising tools to reconstitute detergent-purified MPs in a lipid environment for downstream biochemical, biophysical, and structural characterization (Denisov and Sligar, 2016; Puthenveetil et al., 2017). Recent improvements (Hagn et al., 2013; Nasr et al., 2017), including engineering different versions of the MSP, made it possible to obtain more homogeneous and stable preparations of NDs covering a wide range of sizes (from 4 to 80 nm). Yet, the optimization process to reconstitute individual MPs is still laborious. Indeed, the variant of MSP to be used is highly determined by the shape and the size of the MP to be reconstituted. This implies that different MPs need different MSPs and ND preparations.

Building upon the basic ND idea, a new approach facilitating the reconstitution of MPs in aqueous solution for structural and functional studies has recently been described (Frauenfeld et al., 2016; Lyons et al., 2017), making use of the lipid-binding protein saposin, which forms disc-like particles in the presence of lipids, termed saposin-derived lipid nanoparticles (SapNPs). Here we aimed to identify the most crucial parameters for SapNP formation and the MP reconstitution process by screening for lipid content, various saposin homologs, and ratios thereof. We show that different saposin proteins have different lipid specificities for SapNP formation, with SapA being the least specific. Analytical size-exclusion chromatography (SEC) and small-angle X-ray scattering (SAXS) data show that the SapNPs have a typical lipid-bilayer particle structure and are modular with a positive correlation between their size and the lipid-to-saposin molar ratio. To study more the key parameters influencing the reconstitution process of MPs into SapNPs, we reconstituted four MPs containing 14 to 56 transmembrane α helices by systematically screening various lipids and a broad range of MP-to-lipid-to-saposin molar ratios. All four MPs could be properly reconstituted using only SapA in combination with a variety of lipids and were able to bind their respective ligands, as evidenced by ligand-binding assays. Furthermore, reconstituted MPs were more stable against heat denaturation compared with their n-dodecyl-β-D-maltopyranoside (DDM) detergent-solubilized counterparts. Due to the modular nature of the presented technology, the optimization of the reconstitution process for individual MPs is rather straightforward. We describe a time- and cost-efficient setup to fine-tune the amounts of lipids and saposin required for optimal encapsulation. Finally, analytical SEC and SAXS data collected on two MPs, either solubilized in the detergent DDM or reconstituted in SapA/1-palmitoyl-2-oleoyl-*sn*-glycero-3-phospho-L-serine (POPS) nanoparticles, support the hypothesis that the SapNPs compactly encircle MPs with a minimal number of lipids trapped between the saposin monomers and the MP.

RESULTS AND DISCUSSION

SapNP Formation by Different Saposins and Lipids

Recent studies (Popovic et al., 2012; Frauenfeld et al., 2016; Li et al., 2016) have shown that in the presence of liposomes or detergent-solubilized lipids and within a pH range of 4.5–7.5, SapA undergoes large conformational changes and self-assembles into SapNPs. These studies included only one member of the saposin protein family and were restricted to only a few lipids. The four known human saposin proteins (A, B, C, and D) are the cleavage products of the same Prosaposin precursor protein with an approximate size of 80 residues and a conserved helical fold. To assess the ability of the four different saposin proteins to form SapNPs, we screened them against a library of 17 different detergent-solubilized lipids and lipid mixtures, using analytical SEC. All saposin/lipid pairs were run in duplicates and showed reproducible elution profiles. Based on the screening data we observed different types of behavior: (1) for all favorable saposin/lipid pairs, the free saposin signal declined after addition of the lipid and a second peak, shifted toward lower retention volumes, appeared, indicating the formation of SapNPs. Two examples of such a behavior are shown in Figures 1A and 1B for SapA/18:2 1,2-dilinoleoyl-*sn*-glycero-3-phospho-(1′-*rac*-glycerol) (PG) and SapD/1-myristoyl-2-palmitoyl-*sn*-glycero-3-phosphocholine (MPPC), respectively. (2) In the case of an unfavorable saposin/lipid pair (Figure 1C), we observed an increase in the fluorescence signal of the saposin peak with marginal or no shift. This increase in the intrinsic protein fluorescence signal is an indication that the environment in the vicinity of tyrosine and tryptophan residues has become more hydrophobic, pointing toward saposin/lipid interactions. However, despite these saposin/lipid interactions, we observed only a small or no shift in the elution profile compared to the free saposin peak, attesting the absence of nanoparticle formation. (3) Some of the saposin/lipid pairs form nanoparticles only above a certain threshold of a particular lipid-to-saposin ratio. An example of such a case is SapB/POPS (Figure 1D), where the formation of SapNPs is observed only at a lipid-to-saposin molar ratio of 12 or above. Typically, as in the first case, most of the saposin molecules, if not all, were converted to nanoparticles at a lipid-to-saposin molar ratio of 12. Considering that the area under the saposin monomer peak is directly proportional to the amount of free saposin, we used its ratio in absence and presence of different amounts of lipids to assess the efficiency of a certain saposin/lipid pair for SapNP formation. In the cases of unfavorable saposin/lipid pairs, where the respective peak was not clear, the efficiency of SapNP formation could not be properly estimated and was set to zero. The results for all saposin/lipid pairs, at two different lipid-to-saposin molar ratios (6 and 12), are shown in the form of heatmaps in Figures 1E and 1F. These results support differential lipid specificity for each saposin protein that impacts upon their SapNP formation behavior. The two sphingolipids C18 β-D-lactosyl and galactosyl ceramide and the polyunsaturated 18:2 1,2-dilinoleoyl-*sn*-glycero-3-phosphoethanolamine (PE) are three examples showing SapNP formation with SapA but not with SapB, C, or D (Figures 1E and 1F). SapC and D exhibited a slight preference for lipids with a phosphatidic acid head group (i.e., 1-palmitoyl-2-oleoyl-*sn*-glycero-3-phosphate [POPA] and 18:2 1,2-dilinoleoyl-*sn*-glycero-3-phosphate [PA]). Except for 1-palmitoyl-2-oleoyl-*sn*-glycero-3-phosphoethanolamine (POPE), where nanoparticle formation was observed only at a lipid-to-saposin ratio of 25 or above, SapB was able to efficiently form SapNPs with all phospholipids having mixed acyl chain lengths with one unsaturated

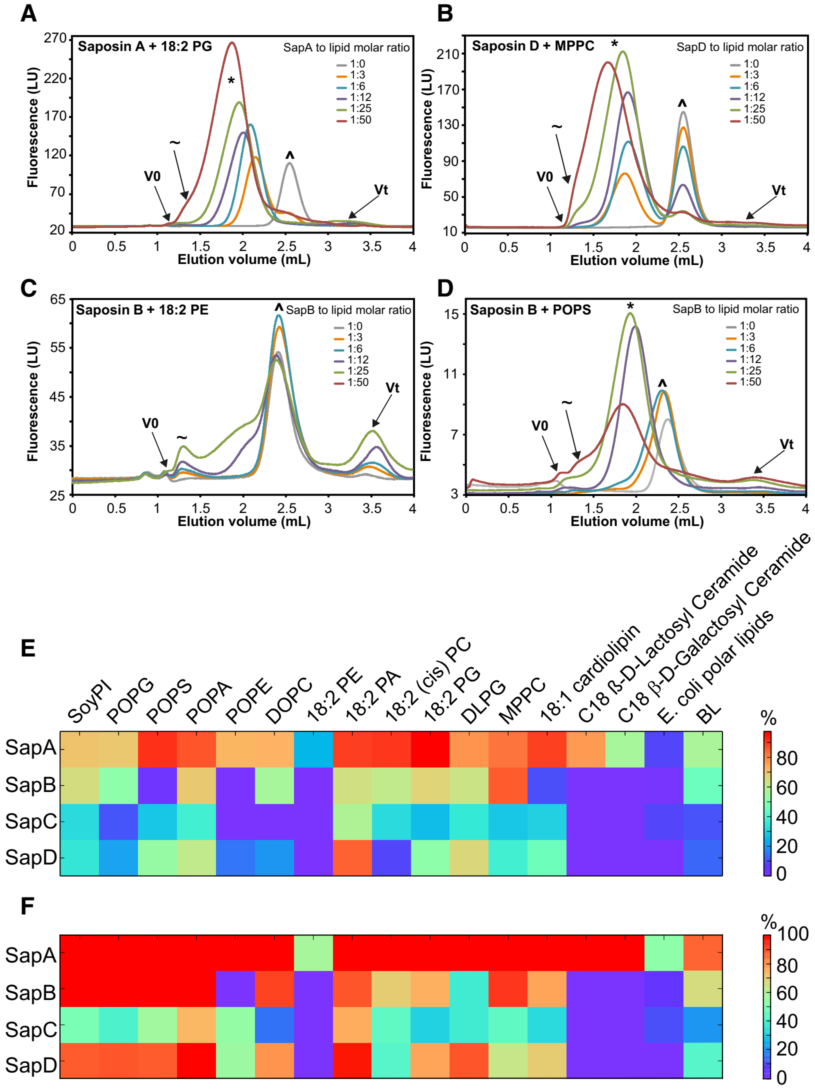

Figure 1. Lipid Specificity of Saposins

(A–D) Gel-filtration chromatograms of SapA/18:2 PG (A), SapD/MPPC (B), SapB/18:2 PE (C), and SapB/POPS (D). The various saposin:lipid molar ratios are color coded as indicated in the figure. The free saposin peak is marked with (^), the SapNPs are marked with (*), and the liposomes/protein aggregates with (~). The void volumes (V0) and the total volumes (Vt) of the columns used are marked.

(E and F) Heatmaps illustrating the efficiency of different Sap/lipid pairs to form SapNPs. Bars on the right show the percentage of saposin converted to SapNPs at a lipid-to-saposin molar ratio of 6 (E) and 12 (F).

fatty acid chain (i.e., 1-palmitoyl-2-oleoyl-*sn*-glycero-3-phospho-(1′-*rac*-glycerol) (POPG), POPS, POPA, and L-α-phosphatidylinositol, Soy [SoyPI]), but was less favorable for polyunsaturated phospholipids. According to our screening data, SapA is the least lipid specific of the four members of the human saposin family. Indeed, under the above-described experimental conditions, SapA was able to form SapNPs with ~90% of the lipids used in this study (Figure 1F). In addition, the recombinant production and purification of SapA are straightforward; the protein is robust and can be stored frozen for months. This, together with its low lipid specificity for SapNP formation, makes SapA the first choice for MP reconstitution into SapNPs.

Characterization of SapNPs in Solution

In order to gain more insights into the properties and physical stability of SapNPs, we further analyzed the analytical SEC data. Our results indicate that the SapNPs contain at least six lipid molecules per saposin. Only a couple of saposin/lipid pairs are 100% efficient at a lipid-to-saposin ratio of 6 (Figure 1E). These observations correlate with recent mass spectrometry data (Li et al., 2016) showing that, in the presence of 1-palmitoyl-2-oleoyl-sn-glycero-3-phosphocholine (POPC) liposomes and at pH 6.8, SapA forms a mixture of SapNPs, composed of SapA tetramers and trimers with 37–60 and 29–36 POPC molecules, respectively. The reported average molecular weights of these SapA/POPC nanoparticles, with sizes of 51 and 68 kDa, are in good agreement with the SEC-estimated apparent molecular weights of 50, 60, and 64 kDa, calculated for three SapA/lipid systems (18:2 PG, POPS, and 1,2-dioleoyl-sn-glycero-3-phosphocholine [DOPC], respectively), at a lipid-to-SapA molar ratio of 12 (Figure S1A). Still, these molecular weight estimates should not be considered as an axiom. In fact, in the cases of favorable saposin/lipid pairs, gel-filtration profiles showed a positive correlation between the apparent molecular weight of the nanoparticles, the broadness of the peak, and the lipid-to-saposin molar ratio (Figures 1A and S1A). An exception to this rule was observed for all four saposins with the lipid MPPC, where the size of the nanoparticles formed with a given saposin remained relatively stable at lipid-to-saposin ratios between 3 and 25 (Figure 1B). This correlation suggests that, unlike NDs, where the length of the MSP determines the diameter of the particles (Denisov et al., 2004), SapNPs are of a modular nature independent of the saposin protein used to form them. The process of self-assembly of the SapNPs primarily depends on the stoichiometric ratios of the lipid and protein components. Adding increasing amounts of lipids in relation to saposin leads to a polydisperse system of SapNPs, and by consequence to a broad molecular weight distribution. Moreover, we monitored the stability over time of two SapA/lipid pairs (SoyPI and POPG at a lipid-to-SapA molar ratio of 12) by analyzing their SEC behavior. As seen from the superposition of the chromatograms after different incubation times (Figures S1B and S1C), the retention volume, for both systems, remained unchanged. Based on the calculated areas under the peaks at different time points, only 5.0% (SapA/SoyPI) and 5.7% (SapA/POPG) of nanoparticles are lost, as attested by the appearance of rather minor aggregation peaks, after 96 hr at 10°C. These results show that, at a specific lipid-to-saposin

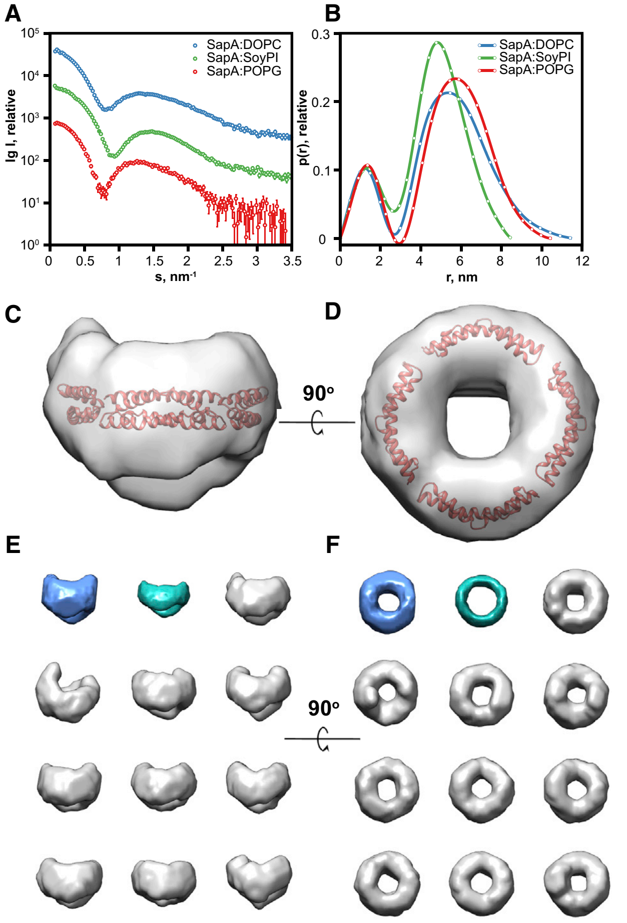

Figure 2. SAXS Data of Empty SapNPs

(A and B) Scattering curves (A) and derived *p(r)* functions (B). The different samples are color coded as indicated in the figure. All nanoparticles were prepared with a lipid-to-SapA molar ratio of 12.

(C–F) The *ab initio* model generated for SapA/DOPC with four SapA molecules (PDB: 4DDJ) fitted in the shape volume (C and D). Ten independent reconstructions were generated using DAMMIF (E and F, in gray) and the program DAMAVER was used to generate the average representative models (E and F, average model in blue and volume-filtered model in cyan).

molar ratio and under the conditions described in this work, SapNPs remain stable and their apparent molecular weights, and by consequence their composition, remain unchanged within at least 5 days of preparation. These findings are in correlation with recent data (Henry Chien et al., 2017) showing that empty SapNPs and SapNP-reconstituted MPs are stable over extended periods of time (up to 4 months).

To obtain more structural information, we measured SAXS on three systems either with serial dilutions in a batch mode, for SapA/DOPC and SapA/SoyPI, or as inline SEC-SAXS for the SapA/DOPC, SapA/SoyPI, and SapA/POPG pairs. Only SapA/SoyPI exhibited small concentration-dependent inter-particle repulsion in the concentration range used for this experiment. An example of the experimental scattering curves with the corresponding normalized pair-distance distribution functions *p(r)* is shown in Figures 2A and 2B. Details of SAXS data collection and derived parameters are shown in Table S3. The R_g values (in Å), estimated from the Guinier analysis of the linear region of the SAXS scattering curve at very small scattering angles, and the *p(r)*-derived maximum particle dimension D_{max} (in Å) are 41.2 ± 0.1 and 115 ± 5, 40.5 ± 0.2 and 105 ± 5, and 35.9 ± 0.2 and 85 ± 4, for SapA/DOPC, SapA/POPG, and SapA/SoyPI, respectively. The observed size differences further reveal the modular nature of these nanoparticles as SapA/SoyPI nanoparticles are smaller than the SapA/DOPC and SapA/POPG nanoparticles despite using the same stoichiometric ratio. These data further support the hypothesis that the size of the SapNPs is dependent on the amount and type of lipids or lipid mixtures used to form them. This change in size can be explained by the difference in the surface area of different lipids.

Overall, the scattering curves obtained for all three systems have similar shapes, with a steep minimum at $s < 1$ nm^{-1} and a broad maximum at $1 < s < 1.5$ nm^{-1} (Figure 2A). This shape is characteristic of a system with a lipid-bilayer core and has been previously reported for a number of discoidal systems including bicelles and NDs (Svergun, 1992; Shih et al., 2005; Skar-Gislinge and Arleth, 2011). All the *p(r)* functions, which reflect the distribution of the components inside the particle, are similar to each other (Figure 2B), indicating a comparable distribution of electron densities for all three SapNPs. These functions are typical of a complex multicontrast system (Feigin and Svergun, 1987). Indeed, the hydrocarbon chains of the lipids, having a lower electron density (~288 e/nm^3) than the aqueous buffer (~334 e/nm^3), negatively contribute to the overall scattering length density, leading to a minimum of the *p(r)* functions close to 3 nm. The SapA and lipid head groups, both having higher electron density (~420 e/nm^3) than the surrounding buffer, give rise to the maxima (Figure 2B). This has been well documented in the case of the similar system of ellipsoidal NDs formed by di-lauroyl-phosphatidylcholine NDs (Skar-Gislinge and Arleth, 2011), where two MSPs encircle a phospholipid bilayer core.

To gain additional insights into the shape and composition of unloaded SapNPs, low-resolution models were reconstructed from the low-s region ($s < 1.0$ nm^{-1}) of the experimental SAXS data *ab initio*, minimizing the contribution from the inhomogeneous internal structure and describing only the overall particle shape/dimensions. The models generated have a circular and somewhat concave discoidal geometry with a hollow core that corresponds to a region of negative contrast (i.e., lower electron density than the solvent), expected for phospholipid hydrocarbon tails of a membrane bilayer (Figures 2C–2F). Model dimensions (D_{max} ~100 Å) and volumes are consistent with SapNPs composed of a phospholipid bilayer core encircled by four or five saposin monomers (Figures 2C and 2D). Altogether, these results provide experimental evidence that the SapNPs have an ND-like structure with the saposin protein and the lipid head groups constituting the shell of the particles and the acyl chains of the lipids the core.

Considerations for Membrane Protein Reconstitution Using SapNPs

The small-conductance mechanosensitive channel T2 from the archaea *Thermoplasma volcanium* and the bacterial peptide transporter PepT$_{So2}$ have recently been reconstituted in a brain lipid mixture using the saposin technology (Frauenfeld et al.,

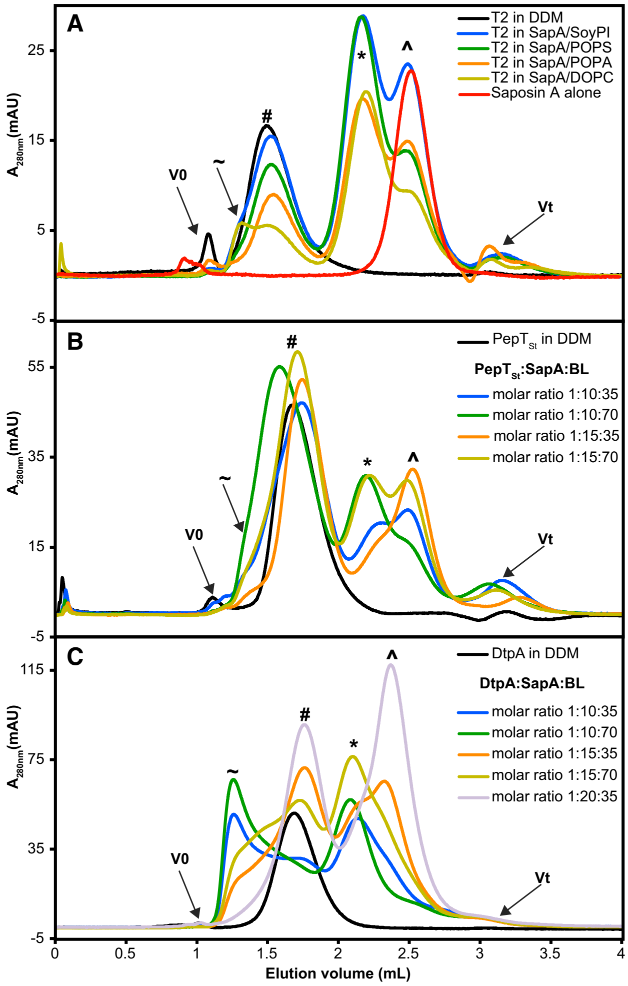

Figure 3. Reconstitution of MPs into SapNPs

(A) Gel-filtration elution profiles of the T2 channel reconstituted in four different SapA/lipid systems as indicated. The T2:SapA:lipid molar ratio is 1:10:40.

(B and C) Gel-filtration chromatograms of PepT$_{St}$ (B) and DtpA (C), reconstituted in SapA/BL nanoparticles.

The various MP-to-SapA-to-lipid stoichiometric ratios are color coded as indicated. The free SapA peak is marked with (^), the empty nanoparticles are marked with (*), the reconstituted membrane proteins with (#), and the soluble aggregates with (~). The black arrows represent the void (V0) and the total (Vt) volumes of the different columns used. Reference elution profiles of DDM-solubilized proteins (black chromatograms) and purified saposin A (red chromatogram in A) are shown.

2016). This work, however, did not include a systematic study highlighting the cardinal parameters governing the reconstitution process. Here we present three case studies for the optimization of MP reconstitution by systematically screening for lipids and lipid mixtures as well as a wide range of MP:saposin:lipid molar ratios (Table S2). All the proteins used in this study were initially purified in the detergent DDM and vary in size and topology. The integral membrane peptide transporters PepT$_{St}$ (52.7 kDa) from *Streptococcus thermophilus* and DtpA (53.9 kDa) from *Escherichia coli* are both composed of 14 transmembrane α helices. It has been shown that PepT$_{St}$ has a dimeric nature in DDM (Quistgaard et al., 2017), while DtpA is purified as a monomer (Weitz et al., 2007). Based on chemical cross-linking experiments, the T2 channel (32.8 kDa per monomer with four transmembrane α helices and a large soluble domain) has been previously reported to be a homopentamer (Löw et al., 2013a). However, in accordance with other bacterial homologs (Wang et al., 2008; Pilotas et al., 2012, 2015), the molecular weight of the T2 channel calculated with triple-detection SEC conjugate analysis is 235 kDa (±0.066%) (Figure S2), which, compared with the sequence-based molecular weight of 230 kDa, suggests a heptameric assembly.

The reconstituted proteins were subjected to a gel-filtration step and analyzed using absorbance at 280 nm and intrinsic protein fluorescence. For MPs that are properly inserted into SapNPs, we observed that the elution peak is shifted only marginally, if at all, towards higher retention volumes compared with the DDM-solubilized protein. On the opposite, an unfavorable reconstitution condition caused the formation of soluble aggregates, which elute near the void volume of the column (Figure 3).

The T2 channel has been reconstituted in four different lipids (SoyPI, POPA, POPS, and DOPC) and exhibited a lipid-dependent behavior. We noticed the appearance of soluble aggregates eluting as a second peak, almost indistinguishable from the void volume, in the case of DOPC and as a small shoulder in the case of the lipid mixture SoyPI. In contrast, using the same stoichiometric ratios, the T2 channel could homogeneously be reconstituted using POPA or POPS and eluted as a single symmetric peak (Figure 3A). The same behavior was observed for all SapA-to-T2 ratios tested.

In the case of the peptide transporter PepT$_{St}$, which was reconstituted in three different lipids (brain total lipid extract [porcine, BL], POPA, and POPS), at a ratio of 10 SapA molecules and 70 BL per PepT$_{St}$ monomer, the peak of the SapNP-reconstituted protein shifted toward lower retention volumes. In addition, compared with other ratios, the peak of the reconstituted material is broader and a small shoulder of soluble aggregates becomes apparent (Figure 3B, green chromatogram), indicative for a heterogeneous sample. On the opposite, a PepT$_{St}$:SapA:BL molar ratio of 1:15:35 resulted in a narrow and symmetric peak distribution (Figure 3B, orange chromatogram), attesting a homogeneous reconstitution. Reconstitution experiments for PepT$_{St}$ in POPA and POPS yielded similar results.

The impact of a balanced saposin:lipid ratio is even more pronounced for the protein DtpA, where a clear relationship between the efficiency of the MP reconstitution and the relative amounts of lipid and SapA is detectable. DtpA was reconstituted in three different lipids (BL, POPA, and POPS). As seen in Figure 3C (green chromatogram), a DtpA:SapA:BL molar ratio of 1:10:70 induced the clustering of the protein, which mainly eluted as soluble aggregates. The signal of the latter peak gradually decreases—along with the empty SapNP peak—by decreasing the amounts of BL and increasing the number of SapA molecules, giving rise to a single peak of properly reconstituted DtpA at a 1:20:35 molar ratio (Figure 3C, light purple chromatogram). Comparable observations were made for all lipids used to reconstitute DtpA. The assignment of the chromatographic peaks has been confirmed by SDS-PAGE (Figure S3) as well as by reference elution profiles of DDM-solubilized proteins and purified SapA (Figure 3).

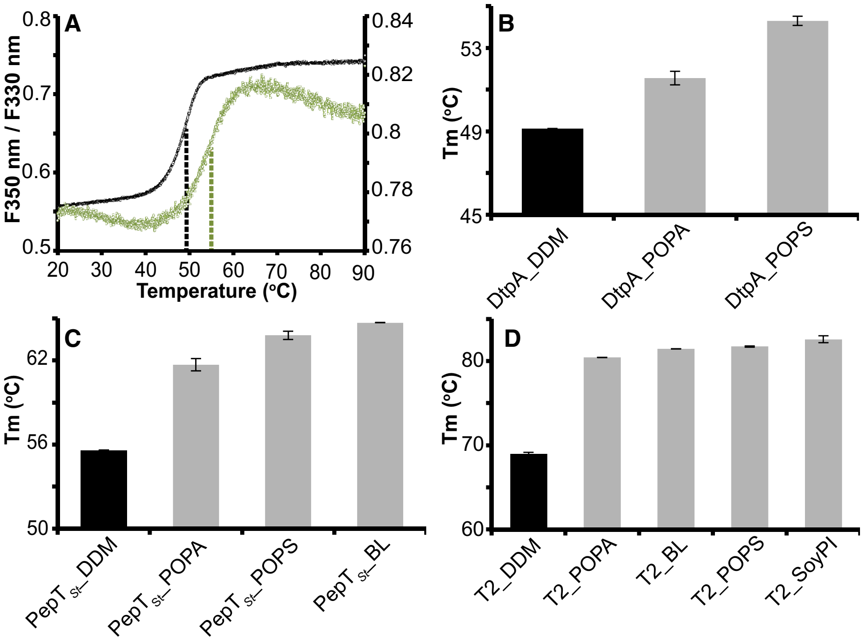

Figure 4. Thermal Stabilization of SapNP-reconstituted MPs

(A) An example of the experimental melting curves obtained with dye-free nanoDSF. The melting temperature (T_m) is derived from the first derivative of the ratio of intrinsic fluorescence intensities recorded at 350 and 330 nm (F350nm/F330nm). The temperature-dependent unfolding of DDM-solubilized DtpA is shown in black with its corresponding y axis on the left and DtpA reconstituted in SapA/POPS in green, with its corresponding y axis on the right. The derived T_m values are marked with dashed lines.

(B–D) Plotted T_m values for DtpA (B), $PepT_{St}$ (C), and T2 (D). The error bars represent standard errors determined from triplicate measurements for each sample. Only SapA was used for the reconstitutions. The lipids used are indicated on the x axes.

Obtaining conditions to reconstitute an MP into SapNPs can be cost and time efficient using small-scale screening and the setup described above. Although the self-assembly process of MPs into SapNPs is still poorly characterized on a molecular level, our extensive screening data allowed us to dissect the reconstitution process and to acquire an in-depth understanding of the essential parameters mediating this process. The lipid-dependent behavior of the T2 channel suggests that the choice of the lipid or lipid mixture is an important protein-dependent factor for successful incorporation. One can heed any available experimental data on the lipid requirement of an MP to choose the lipid or lipid mixture to be tested. Alternatively, screening a lipid mixture such as brain lipid extract and three individual phospholipids, e.g., POPC, POPS, and POPA, is a good start. However, a more critical aspect to consider when using the SapNP technique is the optimization of the MP-to-lipid-to-SapA stoichiometry. Our results show that an excess of lipids while not having sufficient amounts of SapA impedes the reconstitution process. This is, most likely, due to the fact that SapA is in equilibrium between the re-lipidated MP and the available free lipid molecules. We speculate that after re-lipidation of the MP, the free lipid molecules in solution will have a sink effect on SapA and, when in excess, the equilibrium is shifted toward the formation of empty nanoparticles, leading to the aggregation of the MP because of its deprivation of the supply of SapA needed for proper reconstitution. Thus, using a sufficient amount of lipid molecules and an excess of saposin is necessary to prevent the aggregation of the MP and promote its appropriate reconstitution. The transmembrane cross section of the protein of interest, and hence its oligomerization state, most probably dictates the amount of saposin and lipid to use. Independent of the oligomerization state, for MPs in the range of 30–60 kDa, we propose screening a combination of 10–20 saposin and 20–40 lipid molecules per MP monomer. Unlike conventional NDs, where the size of the particles depends on the length of the two encircling amphipathic MSPs and a defined lipid-to-MSP ratio, and where different constructs need to be used in order to adapt to MPs of different topologies and membrane domain cross-sectional areas (Ritchie et al., 2009; Bayburt and Sligar, 2010; Hagn et al., 2013), SapA can form lipid-bilayer-like nanoparticles of different sizes and compositions (Popovic et al., 2012; Li et al., 2016) depending mainly on the lipid-to-SapA ratio. Moreover, using a single SapA construct in combination with various lipids, we could reconstitute several MPs of different sizes and architectures (from 14 to 56 transmembrane α helices, Figure 3 and Frauenfeld et al., 2016).

Optimization of the Reconstitution Process

An important part of the reconstitution process of an MP is the removal of detergent. For traditional reconstitution methods such as the phospholipid bilayer NDs (Ritchie et al., 2009) or amphipols (Zoonens et al., 2014) this is achieved in various ways, e.g., by dilution or dialysis, but mostly by adsorption of polystyrene beads. Until now, removing the detergent using the SapNP technique was done by diluting the reconstituted protein to reach a final detergent concentration below the critical micelle concentration, followed by a final SEC purification step. In an attempt to optimize and simplify the reconstitution process, we examined the possibility of removing the excess of detergent using adsorbent bio-beads or dialysis to replace the dilution step. For this, the T2 channel was reconstituted in SapA/POPA and the detergent removal was carried out using the three different techniques mentioned above. Independent of the detergent removal method used, the mechanosensitive T2 channel was equally well reconstituted (Figure S4). None of the alternative approaches hampered the proper encapsulation of the protein as suggested by the absence of an aggregation peak. Hence, it is possible to remove the excess of detergent from the SapNP-reconstituted MPs using either bio-beads or dialysis instead of dilution. These alternative approaches do not hinder the reconstitution process and are more pragmatic in particular for large-scale reconstitutions.

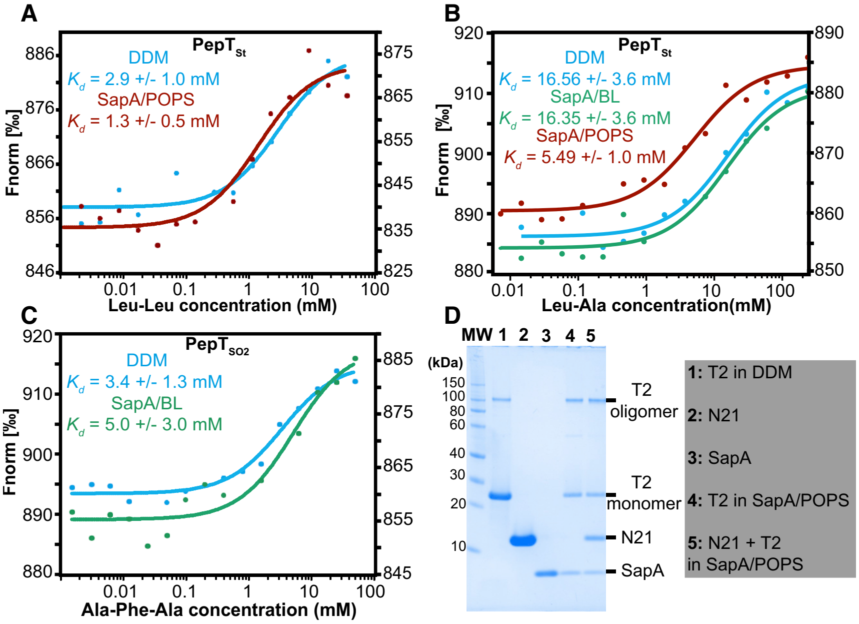

Figure 5. Functional Properties of MPs Reconstituted in SapNPs

(A–C) MST measurements of the binding of Leu-Leu (A) and Leu-Ala (B) dipeptides to PepT$_{St}$ and of Ala-Phe-Ala tripeptide to PepT$_{So2}$ (C). The proteins in DDM or SapNPs are color coded as indicated in the figure. All proteins show typical sigmoidal binding curves and the K_D values estimated from the non-linear fitting (solid lines) of the experimental data (dots) are shown.

(D) SDS-PAGE analysis of the binding of the conformational nanobody 21 (N21) to the SapA/POPS-reconstituted T2 channel. All proteins are labeled and individual purified proteins (lane 1 to 3) are loaded as references. Molecular weight protein markers (MW) are shown.

Thermal Stability and Functional Properties of MPs in SapNPs

Reconstituting MPs in SapNPs has the major advantage of keeping them soluble in a lipid environment in the absence of detergent micelles. However, proteins trapped in SapNPs should also be stable, properly folded, and active. In order to assess the stability of reconstituted proteins, heat denaturation of DDM-solubilized and SapNP-reconstituted DtpA, PepT$_{St}$, and T2 was followed using the dye-free nano differential scanning fluorimeter (nanoDSF) method (Quistgaard et al., 2017). All SapNP-reconstituted proteins were significantly more heat stable and had higher T_m values than in detergent (Figure 4). The observed increase in the T_m values could be confidently assigned to the stability increase of the MP/saposin A complex since saposin A is highly thermostable and does not show an unfolding transition under the experimental conditions. Indeed, the purification of this protein includes a heat precipitation step at 80°C, illustrating the high stability of the protein (see STAR Methods). The fluorescence signal of a saposin A-only sample is temperature dependent as expected but does not show any obvious transition (Figure S5A). An example of the experimentally derived unfolding curves is shown in Figure 4A. The highest stabilization effect was observed in the case the T2 channel reconstituted in SapA/SoyPl, which was ~14°C more stable than the DDM-solubilized protein (Figure 4D). It is worth mentioning that, in all three cases, the lipid POPA had the smallest stabilization effect compared with other lipids. Likewise, it has been reported that PepT$_{So2}$ encapsulated in SapA/BL nanoparticles was more stable (T_m = 72°C) than in the detergent nonyl-β-D-maltopyranoside (T_m = 43°C) (Frauenfeld et al., 2016).

To examine if the proteins encapsulated in SapNPs are active and properly folded, we performed ligand-binding assays using microscale thermophoresis (MST) or analytical SEC followed by SDS-PAGE. Di- and tripeptide binding in solution was used to assess the ligand-binding ability of the SapNP-reconstituted proton-dependent oligopeptide transporters PepT$_{St}$ and PepT$_{So2}$ and compared with protein preparations in DDM. PepT$_{St}$ in SapA/BL and SapA/POPS was able to bind the dipeptide Leu-Leu (Figure 5A) and Leu-Ala (Figure 5B) with estimated K_D values in the millimolar range in agreement with K_D values obtained for the protein in the presence of DDM. Notice that PepT$_{St}$ in SapA/POPS binds Leu-Ala with a slightly higher affinity (Figure 5B, K_D = 5.49 ± 1.0 mM) than in SapA/BL or in DDM (K_D = 16.35 ± 3.6 mM and 16.56 ± 3.6 mM, respectively). In the same manner, PepT$_{So2}$ reconstituted in SapA/BL was able to bind the tripeptide Ala-Phe-Ala with an estimated K_D of 5.0 ± 3.0 mM compared with 3.4 ± 1.3 mM for the protein in DDM (Figure 5C). Given that the measured affinities are quite low and to exclude any unspecific saposin-peptide or lipid-peptide binding, we conducted the same measurements using SapA/BL and SapA/POPS nanoparticles with Leu-Leu and Leu-Ala, respectively. As seen in Figure S5B, no unspecific peptide binding could be observed with empty SapNPs. Last, the folding state of T2 was evaluated using a conformational nanobody, termed N21 (Löw et al., 2013a). Therefore, the reconstituted T2 channel was incubated with isolated N21 and then subjected to a SEC step. The fact that N21 coeluted with reconstituted T2 (Figure 5D, lane 5) confirmed that T2 maintains its conformation after the reconstitution process. In summary, these data support the fact that MPs reconstituted in SapNPs are properly folded, able to bind ligands, and more resistant to heat unfolding than in the presence of detergents. This is in agreement with data obtained for NDs (Laursen et al., 2014; Lamichhane et al., 2015; Ashok and Jaakola, 2016), SMALPs (Long et al., 2013; Dörr et al., 2014), and amphipols (Tifrea et al., 2011; Dahmane et al., 2013; Kleinschmidt and Popot, 2014).

Structural Characterization of SapNP-Reconstituted Membrane Proteins

To characterize in more detail the structures of SapNP-inserted MPs and compare them with the DDM-solubilized MPs, we measured SAXS on the T2 channel and DtpA, either in DDM or in SapNPs. Details of SAXS data collection and derived parameters are shown in Table S4. An example of the experimental scattering curves with the corresponding normalized pair-distance distribution functions *p(r)* is shown in Figures 6A and 6B.

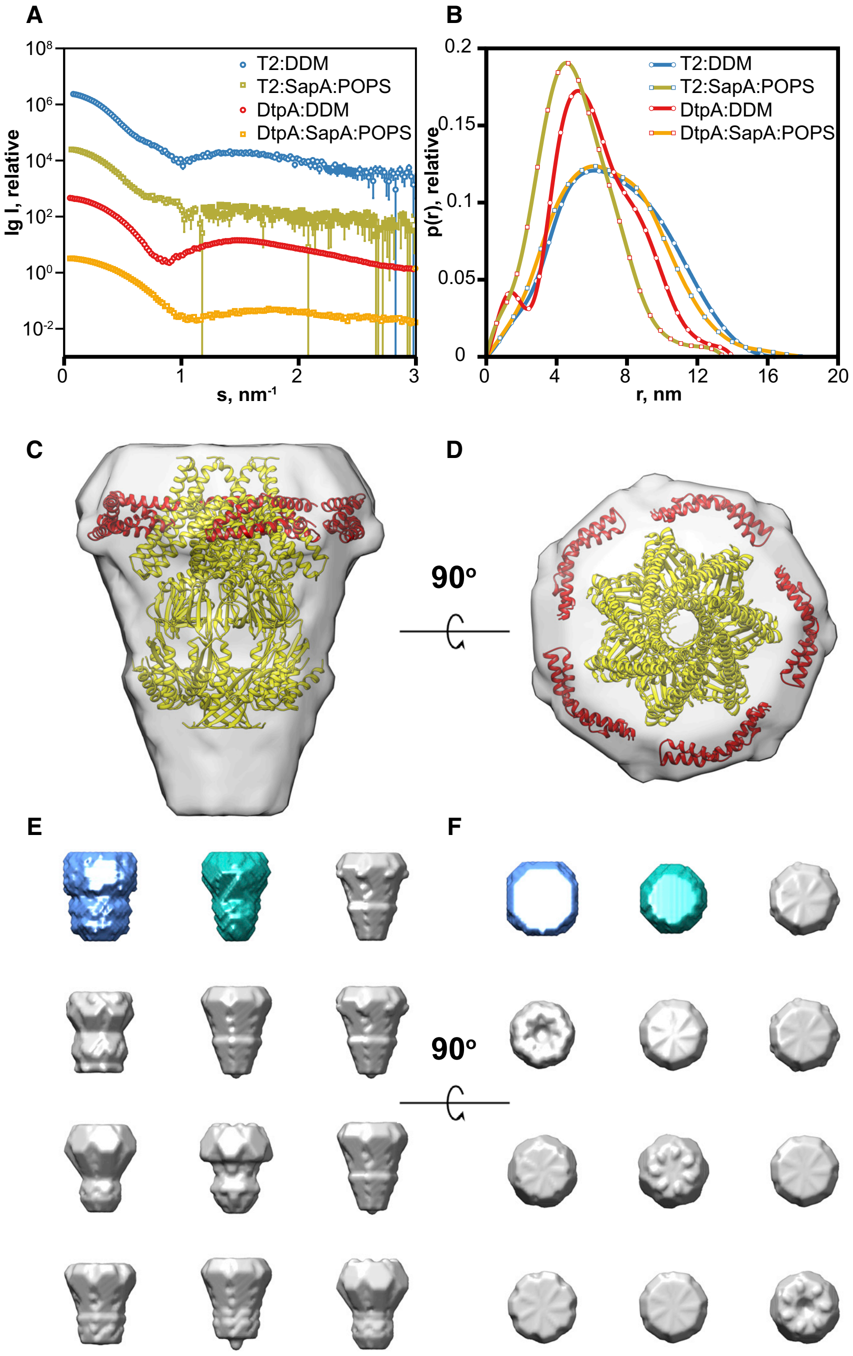

Figure 6. SAXS Data of T2 and DtpA

(A and B) Scattering curves (A) and derived *p(r)* functions (B). The different protein samples, either in DDM or reconstituted in SapA/POPS, are color coded as indicated.

(C–F) *Ab initio* model generated for the T2 channel in SapA/POPS with the crystal structure of a homologous channel from *T. tengcongensis* (PDB: 3T9N) and five SapA molecules (PDB: 4DDJ) fitted in the shape volume (C and D). Ten independent reconstructions were generated using DAMMIF (E and F, in gray) and the program DAMAVER was used to generate the average representative models (E and F, average model in blue and volume-filtered model in cyan).

It is clear that MP insertion radically alters the profile of the real-space distance distributions of the SapNPs. The minima are no longer observed, as the inserted protein increases the scattering density of the particle core. Low-resolution *ab initio* models were reconstructed as described for the empty nanoparticles. In the case of the T2 channel, the T2-DDM complex could be separated from the free DDM micelles in solution using SEC (Figure S2). The scattering curves, the *p(r)* functions, and the generated *ab initio* models for the protein in detergent solution or encapsulated in a SapNPs exhibit comparable profiles. Both models have an elongated cone structure (Figures 6C–F and S6A–S6D) with maximum dimensions of 165 and 180 Å, respectively. To assess the agreement of these models with known structural information, the crystal structure of the heptameric mechanosensitive channel protein from *Thermoanaerobacter tengcongensis* (Zhang et al., 2012) was spatially mapped into the *ab initio* shape volumes (Figures 6C, 6D, S6A, and S6B). Topologically the SAXS models are in good agreement with the T2 homologous structure. Strikingly, in the SapNP model, the volume around the transmembrane region of the heptamer, assumedly occupied by the SapA and the lipid molecules, is not larger than the volume occupied by the DDM corona in the DDM model (Figures S6A–S6D) and can accommodate at least five saposin molecules (Figures 6C and 6D).

In the case of DtpA, a 53.9 kDa protein with a transmembrane-only architecture, the scattering curve and the derived *p(r)* function of the protein in DDM are substantially different from that of the SapNP-trapped protein (Figures 6A and 6B). In fact, unlike for the T2 channel, the DDM-DtpA complex could not be readily separated from the free DDM micelles in solution using SEC. This made it very difficult, if not impossible, to extract any useful structural information from the protein in DDM. Indeed, a double-bell-shaped *p(r)* function is observed with a minimum at ~3 nm, consistent with that expected for core-shell micelles (Figure 6B). Thus it is likely that the SAXS signal of DtpA is dominated by free DDM micelles (~72 kDa), which are in the same size range as the protein itself. However, for the detergent-free SapNP-reconstituted DtpA, we could confidently generate an *ab initio* model exhibiting an ellipsoidal geometry (Figures S6E and S6F). In the absence of high-resolution structural information, a homologous structure (PDB: 4Q65) was manually docked into the *ab initio* volume. The most plausible orientation is that where the principal axis of DtpA lies along

the minor axis of the *ab initio* model, yielding an arrangement of one transporter unit with three or four saposin monomers.

In agreement with analytical SEC (Figure 3), the generated *ab initio* models support the hypothesis that SapNPs compactly encircle MPs with a minimal number of lipids trapped between the saposin monomers and the protein. This observation is further supported by the fact that the negative contributions to the *p(r)* functions, due to the hydrocarbon chains of the lipids, cannot be clearly discerned (Figure 6B) for the proteins trapped in SapNPs.

Overall, the data in Figure 6 vividly illustrate the advantage of SapNPs over DDM for SAXS studies. Indeed, the curves recorded with DDM solubilization reveal clear additional minima attributed to the DDM micelles (especially pronounced for DtpA). In contrast, the SapNPs yield scattering data corresponding to overall smaller and more homogeneous particles, and these data can be directly interpreted with *ab initio* analysis.

DISCUSSION

The proposed saposin lipid nanoparticle technology can maintain MP solubility in a lipid environment without detergent. It provides a simple and easy-to-optimize alternative reconstitution strategy to detergent micelles and other scaffold technologies such as amphipols and NDs. Our extensive screening results show that saposins have different specificities for different lipids with regard to SapNP formation. SapA has proven to be the least specific among the members of the human saposin family, which makes it the saposin of choice for MP reconstitution. The SAXS data support a discoidal saposin-derived nanoparticle structure with a lipid-bilayer core. The balance between the amounts of lipids and saposin with respect to the amount of proteins is the most critical aspect to consider when using SapNPs. Several MPs with different topologies and transmembrane cross-sectional areas have been successfully reconstituted in various lipids using a single construct of SapA. Our data indicate that SapNP-encapsulated MPs can bind their respective ligands, as shown for $PepT_{St}$ and $PepT_{So2}$, and are properly folded, as shown for the T2 channel. All SapNP-reconstituted proteins used in this study were thermally more stable than in detergent solution. These observations highlight the advantage of the SapNP technology and promote its high versatility and adaptability, making it a universal tool to encapsulate MPs in a detergent-free lipid environment for biophysical and biochemical studies. Moreover, the saposin lipid nanoparticles provide a powerful tool for structural investigations of MPs (Lyons et al., 2017). Specifically for the SAXS studies, saposins offer a significant advantage over the use of detergent, providing compact and homogeneous particles. This allows for direct *ab initio* shape analysis, and this analysis can further be extended to meaningful rigid body modeling utilizing the known structure of SapA. The single-particle cryoelectron microscopy (cryo-EM) structure of $PepT_{So2}$ in SapA/BL nanoparticles (Frauenfeld et al., 2016), together with the SAXS studies conducted on T2 and DtpA, indicate that SapNPs encircle MPs in a compact way and stabilize the system with a limited number of lipids trapped between the saposin and the MP. The suitability of the technology for solution nuclear magnetic resonance (NMR) studies of MPs has also been shown recently (Henry Chien et al., 2017). It is worth mentioning that minimizing the size of the saposin/lipid “belt” around the MP can be beneficial for different biochemical, biophysical, and structural applications. This lipid-limiting regime can, however, be quite different from a native lipid bilayer. To explore these further it will be necessary to study the structure and function of MPs reconstituted in different sizes of SapNPs. In addition high-resolution structural studies, using cryo-EM, crystallography, or NMR, will be necessary to understand how the saposin molecules wrap around the MPs and how many lipids are trapped between them.

STAR★METHODS

Detailed methods are provided in the online version of this paper and include the following:

- KEY RESOURCES TABLE
- CONTACT FOR REAGENT AND RESOURCE SHARING
- METHOD DETAILS
  - Cloning, Expression and Purification of Saposins
  - Cloning, Expression and Purification of Membrane Proteins
  - Lipid Preparation and Screening for SapNP Formation
  - Apparent Molecular Weight of SapNPs
  - SapNPs Physical Stability Over Time
  - Triple-Detection SEC Conjugate Analyses
  - Reconstitution of Membrane Proteins into SapNPs
  - Reconstitution of the Mechanosensitive Channel into SapNPs Using Bio-Beads and Dialysis
  - Analytical Gel Filtration Setup and Data Analysis
  - Protein Thermal Unfolding and Ligand Binding
  - SAXS Measurements
- DATA AND SOFTWARE AVAILABILITY
  - Software Availability
  - Data Availability

SUPPLEMENTAL INFORMATION

Supplemental Information includes five figures and four tables and can be found with this article online at https://doi.org/10.1016/j.str.2018.01.007.

ACKNOWLEDGMENTS

We thank the Sample Preparation and Characterization Facility of EMBL-HH for support with nanoDSF measurements and Grzegorz Chojnowski for helping with the generation of the heatmaps. This work was funded by the Horizon 2020 Program of the European Union (iNEXT grant, project 653706) and the Swedish Research Council (grant 621-2013-5905).

AUTHOR CONTRIBUTIONS

Conceptualization, A.F. and C.L.; Methodology, A.F., H.D.T.M., D.I.S., and C.L; Investigation, A.F., H.D.T.M., M.M.M., and Y.U.B.; Writing – Review & Editing, A.F., H.D.T.M., D.I.S., and C.L.; Funding Acquisition, D.I.S. and C.L.; Resources, D.I.S. and C.L.; Supervision, D.I.S. and C.L.

DECLARATION OF INTEREST

The authors declare no competing interests.

Received: August 25, 2017
Revised: November 27, 2017
Accepted: January 10, 2018
Published: February 6, 2018

REFERENCES

Abla, M., Unger, S., Keller, S., Bonneté, F., Ebel, C., Pucci, B., Breyton, C., and Durand, G. (2015). Micellar and biochemical properties of a propyl-ended fluorinated surfactant designed for membrane-protein study. J. Colloid. Interface Sci. *445*, 127–136.

Aryal, P., Jarerattanachat, V., Clausen, M.V., Schewe, M., McClenaghan, C., Argent, L., Conrad, L.J., Dong, Y.Y., Pike, A.C.W., Carpenter, E.P., et al. (2017). Bilayer-mediated structural transitions control mechanosensitivity of the TREK-2 K2P channel. Structure *25*, 708–718.

Ashok, Y., and Jaakola, V.P. (2016). Nanodisc-Tm: rapid functional assessment of nanodisc reconstituted membrane proteins by CPM assay. MethodsX *3*, 212–218.

Bayburt, T.H., and Sligar, S.G. (2010). Membrane protein assembly into Nanodiscs. FEBS Lett. *584*, 1721–1727.

Beckham, K.S.H., Ciccarelli, L., Bunduc, C.M., Mertens, H.D.T., Ummels, R., Wolfgang, L., Mayr, J., Rettel, M., Savitski, M.M., Svergun, D., et al. (2017). Structure of the mycobacterial ESX-5 type VII secretion system membrane complex by single-particle analysis. Nat. Microbiol. *2*, 17047.

Blanchet, C.E., Spilotros, A., Schwemmer, F., Graewert, M.A., Kikhney, A., Jeffries, C.M., Franke, D., Mark, D., Zengerle, R., Cipriani, F., et al. (2015). Versatile sample environments and automation for biological solution X-ray scattering experiments at the P12 beamline (PETRA III, DESY). J. Appl. Crystallogr. *48*, 431–443.

Bowie, J.U. (2001). Stabilizing membrane proteins. Curr. Opin. Struct. Biol. *11*, 397–402.

Breyton, C., Pucci, B., and Popot, J.-L. (2010). Amphipols and fluorinated surfactants: two alternatives to detergent for studying membrane proteins. Methods Mol. Biol. *601*, 219–245.

Breyton, C., Gabel, F., Lethier, M., Flayhan, A., Durand, G., Jault, J.-M., Juillan-Binard, C., Imbert, L., Moulin, M., Ravaud, S., et al. (2013a). Small angle neutron scattering for the study of solubilised membrane proteins. Eur. Phys. J. E. Soft Matter *36*, 71.

Breyton, C., Flayhan, A., Gabel, F., Lethier, M., Durand, G., Boulanger, P., Chami, M., and Ebel, C. (2013b). Assessing the conformational changes of pb5, the receptor-binding protein of phage T5, upon binding to its *Escherichia coli* receptor FhuA. J. Biol. Chem. *288*, 30763–30772.

Conde, A., Chaves, M.M., and Gerós, H. (2011). Membrane transport, sensing and signaling in plant adaptation to environmental stress. Plant Cell Physiol *52*, 1583–1602.

Dahmane, T., Rappaport, F., and Popot, J.-L. (2013). Amphipol-assisted folding of bacteriorhodopsin in the presence or absence of lipids: functional consequences. Eur. Biophys. J. *42*, 85–101.

Denisov, I.G., and Sligar, S.G. (2016). Nanodiscs for structural and functional studies of membrane proteins. Nat. Struct. Mol. Biol. *23*, 481–486.

Denisov, I.G., Grinkova, Y.V., Lazarides, A.A., and Sligar, S.G. (2004). Directed self-assembly of monodisperse phospholipid bilayer Nanodiscs with controlled size. J. Am. Chem. Soc. *126*, 3477–3487.

Dörr, J.M., Koorengevel, M.C., Schäfer, M., Prokofyev, A.V., Scheidelaar, S., van der Cruijsen, E.A., Dafforn, T.R., Baldus, M., and Killian, J.A. (2014). Detergent-free isolation, characterization, and functional reconstitution of a tetrameric K+ channel: the power of native nanodiscs. Proc. Natl. Acad. Sci. USA *111*, 18607–18612.

Duquesne, K., and Strugis, J.N. (2010). Membrane protein solubilization. Methods Mol. Biol. *601*, 205–217.

Feigin, L.A., and Svergun, D.I. (1987). Interpretation of scattering by inhomogeneous particles. In Structure Analysis by Small-angle X-ray and Neutron Scattering, G.W. Taylor, ed. (Plenum Press), pp. 107–146.

Flayhan, A., Wien, F., Paternostre, M., Boulanger, P., and Breyton, C. (2012). New insights into pb5, the receptor binding protein of bacteriophage T5, and its interaction with its *Escherichia coli* receptor FhuA. Biochimie *94*, 1982–1989.

Franke, D., and Svergun, D.I. (2009). Dammif, a program for rapid ab-initio shape determination in small-angle scattering. J. Appl. Crystallogr. *42*, 342–346.

Franke, D., Petoukhov, M.V., Konarev, P.V., Panjkovich, A., Tuukkanen, A., Mertens, H.D.T., Kikhney, A.G., Hajizadeh, N.R., Franklin, J.M., Jeffries, C.M., and Svergun, D.I. (2017). ATSAS 2.8: a comprehensive data analysis suite for small-angle scattering from macromolecular solutions. J. Appl. Crystallogr. *50*, 1212–1225.

Frauenfeld, J., Loving, R., Armache, J.-P., Sonnen, A.F.-P., Guettou, F., Moberg, P., Zhu, L., Jegerschold, C., Flayhan, A., Briggs, J.A.G., et al. (2016). A saposin-lipoprotein nanoparticle system for membrane proteins. Nat. Methods *13*, 345–351.

Garavito, R.M., and Fergusson-Miller, S. (2001). Detergents as tools in membrane biochemistry. J. Biol. Chem. *276*, 32403–32406.

Gimpl, K., Klement, J., and Keller, S. (2016). Characterising protein/detergent complexes by triple-detection size-exclusion chromatography. Biol. Proced. Online *18*, 4.

Giuliani, S.E., Frank, A.M., Corgliano, D.M., Seifert, C., Hauser, L., and Collart, F.R. (2011). Environment sensing and response mediated by ABC transporters. BMC Genomics *12* (S1), S8.

Grove, J., and Marsh, M. (2011). The cell biology of receptor-mediated virus entry. J. Cell Biol. *195*, 1071–1082.

Guettou, F., Quistgaard, E.M., Trésaugues, L., Moberg, P., Jegerschöld, C., Zhu, L., Jong, A.J., Nordlund, P., and Löw, C. (2013). Structural insights into substrate recognition in proton-dependent oligopeptide transporters. EMBO Rep. *14*, 804–810.

Hagn, F., Etzkorn, M., Raschle, T., and Wagner, G. (2013). Optimized phospholipid bilayer nanodiscs facilitate high-resolution structure determination of membrane proteins. J. Am. Chem. Soc. *135*, 1919–1925.

Hammarström, M., Woestenenk, E.A., Hellgren, N., Härd, T., and Berglund, H. (2006). Effect of N-terminal solubility enhancing fusion proteins on yield of purified target protein. J. Struct. Funct. Genomics *7*, 1–14.

Haswell, E.S., Phillips, R., and Rees, D.C. (2011). Mechanosensitive channels: what can they do and how do they do it. Structure *19*, 1356–1369.

Henry Chien, C.-T., Helfinger, L.R., Bostock, M.J., Solt, A., Tan, Y.L., and Nietlispach, D. (2017). An adaptable phospholipid membrane mimetic system for solution NMR studies of membrane proteins. J. Am. Chem. Soc. *139*, 14829–14832.

Hunter, J.D. (2007). Matplotlib: a 2D graphics environment. Comput. Sci. Eng. *9*, 90–95.

Kleinschmidt, J.H., and Popot, J.-L. (2014). Folding and stability of integral membrane proteins in amphipols. Arch. Biochem. Biophys. *564*, 327–343.

Knütter, I., Hartrodt, B., Theis, S., Foltz, M., Rastetter, M., Daniel, H., Neubert, K., and Brandsch, M. (2004). Analysis of the transport properties of side chain modified dipeptides at the mammalian peptide transporter PEPT1. Eur. J. Pharm. Sci. *21*, 61–67.

Konarev, P.V., Petoukhov, M.V., and Svergun, D.I. (2016). Rapid automated superposition of shapes and macromolecular models using spherical harmonics. J. Appl. Crystallogr. *49*, 953–960.

Lamichhane, R., Liu, J.J., Pljevaljcic, G., White, K.L., Van der Schans, E., Katritch, V., Stevens, R.C., Wüthrich, K., and Millar, D.P. (2015). Single-molecule view of basal activity and activation mechanisms of the G protein-coupled receptor β2AR. Proc. Natl. Acad. Sci. USA *112*, 14254–14259.

Laursen, T., Singha, A., Rantzau, N., Tutkus, M., Borch, J., Hedegård, P., Stamou, D., Møller, B.L., and Hatzakis, N.S. (2014). Single molecule activity measurements of cytochrome P450 oxidoreductase reveal the existence of two discrete functional states. ACS. Chem. Biol. *9*, 630–634.

Lee, S.C., Knowles, T.J., Postis, V.L., Jamshad, M., Parslow, R.A., Lin, Y.P., Goldman, A., Sridhar, P., Overduin, M., Muench, S.P., and Dafforn, T.R.

(2016). A method for detergent-free isolation of membrane proteins in their local lipid environment. Nat. Protoc. *11*, 1149–1162.

Li, J., Richards, M.R., Bagal, D., Campuzano, I.D., Kitova, E.N., Xiong, Z.J., Privé, G.G., and Klassen, J.S. (2016). Characterizing the size and composition of Saposin A lipoprotein picodiscs. Anal. Chem. *88*, 9524–9531.

Liao, M., Cao, E., Julius, D., and Cheng, Y. (2013). Structure of the TRPV1 ion channel determined by electron cryo-microscopy. Nature *504*, 107–122.

Lodish, H., Berk, A., Zipursky, S.L., Matsudaira, P., Baltimore, D., and Darnell, J. (2000). Cell-cell adhesion and communication. In Molecular Cell Biology, Fourth Edition, H. Lodish, A. Berk, S.L. Zipursky, P. Matsudaira, D. Baltimore, and J. Darnell, eds. (W.H. Freeman), Section 22-1.

Long, A.R., O'Brien, C.C., Mathotra, K., Schwall, C.T., Albert, A.D., Watts, A., and Alder, N.N. (2013). A detergent-free strategy for the reconstitution of active enzyme complexes from native biological membranes into nanoscale discs. BMC Biotechnol. *13*, 41.

Löw, C., Yau, Y.H., Pardon, E., Jegerschöld, C., Wåhlin, L., Quistgaard, E.M., Moberg, P., Geifman-Shochat, S., Steyaert, J., and Nordlund, P. (2013a). Nanobody mediated crystallization of an archeal mechanosensitive channel. PLoS One *8*, e77984.

Löw, C., Moberg, P., Quistgaard, E.M., Hedrén, M., Guettou, F., Frauenfeld, J., Haneskog, L., and Nordlund, P. (2013b). High-throughput analytical gel filtration screening of integral membrane proteins for structural studies. Biochim. Biophys. Acta *1830*, 3497–3508.

Lyons, J.A., Bøggild, A., Nissen, P., and Frauenfeld, J. (2017). Saposin-lipoprotein scaffolds for structure determination of membrane transporters. Methods Enzymol. *594*, 85–99.

Nasr, M.L., Baptista, D., Strauss, M., Sun, Z.Y.J., Grigoriu, S., Huser, S., Pluckthun, A., Hagn, F., Walz, T., Hogle, J.M., and Wagner, G. (2017). Covalently circularized nanodiscs for studying membrane proteins and viral entry. Nat. Methods *14*, 49–52.

Norimastu, Y., Hasegawa, K., Shimizu, N., and Toyoshima, C. (2017). Protein-phospholipid interplay revealed with crystals of a calcium pump. Nature *545*, 193–198.

Osakabe, Y., Yamaguchi-Shinozaki, K., Shinozaki, K., and Tran, L.S. (2013). Sensing the environment: key roles of membrane-localized kinases in plant perception and response to abiotic stress. J. Exp. Bot. *64*, 445–458.

Pilotas, C., Ward, R., Branigan, E., Rasmussen, A., Hagelueken, G., Huang, H., Black, S.S., Both, I.R., Schiemann, O., and Naismith, J.H. (2012). Conformational state of the MscS mechanosensitive channel in solution revealed by pulsed electron-electron double resonance (PELDOR) spectroscopy. Proc. Natl. Acad. Sci. USA *109*, E2675–E2682.

Pilotas, C., Dahl, A.C., Rasmussen, T., Mahendran, K.R., Smith, T.K., Marius, P., Gault, J., Banda, T., Rasmussen, A., Miller, S., et al. (2015). The role of lipids in mechanosensation. Nat. Struct. Mol. Biol. *22*, 991–998.

Popovic, K., Holyake, J., Pomès, R., and Privé, G.G. (2012). Structure of saposin A lipoprotein discs. Proc. Natl. Acad. Sci. USA *109*, 2908–2912.

Puthenveetil, R., Nguyen, K., and Vinogradova, O. (2017). Nanodiscs and solution NMR: preparation, application and challenges. Nanotechnol. Rev. *6*, 111–126.

Quistgaard, E.M., Martinez Molledo, M., and Löw, C. (2017). Structure determination of a major facilitator peptide transporter: inward facing $PepT_{St}$ from *Streptococcus thermophilus* crystallized in space group P3121. PLoS One *12*, e0173126.

Ritchie, T.K., Grinkova, Y.V., Bayburt, T.H., Denisov, I.G., Zolnerciks, J.K., Atkins, W.M., and Sligar, S.G. (2009). Reconstitution of membrane proteins in phospholipid bilayer nanodiscs. Methods Enzymol. *464*, 211–231.

Savitsky, P., Bray, J., Cooper, C.D.O., Marsden, B.D., Mahajan, P., Burgess-Brown, N.A., and Gileadi, O. (2010). High-throughput production of human proteins for crystallization: the SGC experience. J. Struct. Biol. *172*, 3–13.

Shih, A.Y., Denisov, I.G., Phillips, J.C., Sligar, S.G., and Schulten, K. (2005). Molecular dynamics simulations of discoidal bilayers assembled from truncated human lipoproteins. Biophys. J. *88*, 548–556.

Skar-Gislinge, N., and Arleth, L. (2011). Small-angle scattering from phospholipid nanodiscs: derivation and refinement of a molecular constrained analytical model form factor. Phys. Chem. Chem. Phys. *13*, 3161–3170.

Sukharev, S., and Sachs, F. (2012). Molecular force transduction by ion channels: diversity and unifying principles. J. Cell Sci. *125*, 3075–3083.

Svergun, D.I. (1992). Determination of the regularization parameter in indirect-transform methods using perceptual criteria. J. Appl. Crystallogr. *25*, 495–503.

Tifrea, D.F., Sun, G., Pal, S., Zardeneta, G., Cocco, M.J., Popot, J.-L., and de la Maza, L.M. (2011). Amphipols stabilize the *Chlamydia* major outer membrane protein and enhance its protective ability as a vaccine. Vaccine *29*, 4623–4631.

Verkman, A.S., and Mitra, A.K. (2000). Structure and function of aquaporin water channels. Am. J. Physiol. Ren. Physiol *278*, 13–28.

Volkov, V.V., and Svergun, D.I. (2003). Uniqueness of ab initio shape determination in small-angle scattering. J. Appl. Crystallogr. *36*, 860–864.

Wallin, E., and von Heijne, G. (1998). Genome-wide analysis of integral membrane proteins from eubacterial, archaean, and eukaryotic organisms. Protein Sci. *7*, 1029–1038.

Wang, W., Black, S.S., Edwards, M.D., Miller, S., Morisson, E.L., Bartlett, W., Dong, C., Naismith, J.H., and Booth, I.R. (2008). The structure of an open form of an *E. coli* mechanosensitive channel at 3.45 Å resolution. Science *321*, 1179–1183.

Weitz, D., Harder, D., Casagrande, F., Fotiadis, D., Obrdlik, P., Kelety, B., and Daniel, H. (2007). Functional and structural characterization of a prokaryotic peptide transporter with features similar to mammalian PEPT1. J. Biol. Chem. *282*, 2832–2839.

Zhang, X., Wang, J., Feng, Y., Ge, J., Li, W., Sun, W., Iscla, I., Yu, J., Blount, P., Li, Y., and Yang, M. (2012). Structure and molecular mechanism of an anion-selective mechanosensitive channel of small conductance. Proc. Natl. Acad. Sci. USA *109*, 18180–18185.

Zoonens, M., and Popot, J.-L. (2014). Amphipols for each season. J. Membr. Biol. *247*, 759–796.

Zoonens, M., Zito, F., Martinez, K.L., and Popot, J.-L. (2014). Amphipols: a general introduction and some protocols. In Membrane Proteins Production for Structural Analysis, I. Mus-Veteau, ed. (Springer), pp. 173–203.

STAR★METHODS

KEY RESOURCES TABLE

REAGENT or RESOURCE	SOURCE	IDENTIFIER
Chemicals, Peptides, and Recombinant Proteins		
18:1 Cardiolipin	Avanti Polar Lipids, Inc.	710335
SoyPI	Avanti Polar Lipids, Inc.	840044
16:0-18:1 PS (POPS)	Avanti Polar Lipids, Inc.	840034
16:0-18:1 PG (POPG)	Avanti Polar Lipids, Inc.	840457
16:0-18:1 PE (POPE)	Avanti Polar Lipids, Inc.	850757
16:0-18:1 PA (POPA)	Avanti Polar Lipids, Inc.	840857
18:2 PG (poly-unsaturated)	Avanti Polar Lipids, Inc.	840485
18:2 PE (poly-unsaturated)	Avanti Polar Lipids, Inc.	850755
18:2 PA (poly-unsaturated)	Avanti Polar Lipids, Inc.	840885
18:2 (cis) PC (DLPC, poly-unsaturated)	Avanti Polar Lipids, Inc.	850385
14:0-16:0 PC	Avanti Polar Lipids, Inc.	850445
22:0 PC	Avanti Polar Lipids, Inc.	850371
12:0 PG	Avanti Polar Lipids, Inc.	840435
C18 Lactosyl(ß) Ceramide (d18:1/18:0)	Avanti Polar Lipids, Inc.	860598
C18 Galactosyl(ß) Ceramide (d18:1/18:0)	Avanti Polar Lipids, Inc.	860844
Brain total lipid extract	Avanti Polar Lipids, Inc.	131101
E. coli polar lipid extract	Avanti Polar Lipids, Inc.	100600
n-Dodecyl-β-D-Maltopyranoside	Anatrace	D310
n-Dodecyl-N,N-Dimethylamine-N-Oxide	Anatrace	D360
Terrific broth	Melford	T1702
Ampicillin	Carl Roth	HP62
Tetracycline	Carl Roth	0237
Chloramphenicol	Carl Roth	3886
Kanamycin	Carl Roth	T832
Isopropyl-β-D-thiogalactopyranoside	Carl Roth	CN08
Imidazole	Carl Roth	X998
EDTA-free protease inhibitor cocktail	Roche	11 836 170 001
Deposited Data		
SAXS: SaposinA/DOPC	This paper	SASDC27
SAXS: SaposinA/SoyPI	This paper	SASDC37
SAXS: SaposinA/POPG	This paper	SASDC47
SAXS: T2 in SaposinA/POPS	This paper	SASDCX6
SAXS: T2 in DDM	This paper	SASDCY6
SAXS: DtpA in SaposinA/POPS	This paper	SASDCZ6
Bacterial and Virus Strains		
E. coli C41 (DE3)	EMBL-Hamburg	n/a
E. coli Rosetta gami-2 (DE3)	Novagen	71351
Recombinant DNA		
Plasmid: pNIC28-Bsa4	Savitsky et al., 2010	n/a
Plasmid: ptH27	Hammarström et al., 2006	n/a
Other		
Bio-Beads SM-2 Adsorbents	Bio-Rad	1528920
Slide-A-Lyzer G2 Dialysos Cassette	Thermo Fisher Scientific	87723

CONTACT FOR REAGENT AND RESOURCE SHARING

Further information and requests for resources and reagents should be directed to and will be fulfilled by the Lead Contact, Christian Löw (christian.loew@embl-hamburg.de).

METHOD DETAILS

Cloning, Expression and Purification of Saposins

The Prosaposin gene regions coding for saposin A (residues 60 to 140), B (residues 195 to 273), C (residues 311 to 391), and, D (residues 407 to 484), were individually cloned into the pNIC28-Bsa4 vector (Savitsky et al., 2010), which allows the expression of the corresponding protein with an N-terminal tag of 23 residues (MHHHHHHSSGVDLGTENLYFQ*SM) containing a Hexahistidine (His_6) tag and a Tobacco Etch Virus (TEV) protease cleavage site (*) for tag removal. Saposin proteins were overproduced in *E. coli* Rosetta gami-2 (DE3) cells. Cells were grown in terrific broth supplemented with tetracycline, chloramphenicol and kanamycin at 37 °C and induced with 1 mM IPTG at OD_{600nm} between 0.8 and 1. Three to four hours post-induction, the cells were harvested by centrifugation at 6,000 rpm for 20 min, flash-frozen in liquid nitrogen, and stored at -80 °C.

For purification, frozen cell pellets were resuspended (1 gram of cells in 5 mL of buffer) in lysis buffer (20 mM sodium phosphate (Na-P) pH 7.5, 300 mM NaCl, 15 mM imidazole pH 7.5, 5 % glycerol, 5 U/mL DNase, 1 mg/mL lysozyme, and 1 tablet of EDTA-free protease inhibitor cocktail for 50 mL of buffer) and broken by sonication (3 × 3 min, 0.5 s on / 0.5 s off, at 50% intensity). The cell lysate was centrifuged at 4,000 rpm for 30 min to remove unbroken cells. The supernatant was heated for 10 min at 80 °C and then subjected to a second centrifugation step at 20,000 rpm for 45 min. A first immobilized metal affinity chromatography (IMAC) step was carried out by incubating the supernatant with the IMAC resin (2 mL of resin for 50 mL of lysate), pre-equilibrated with the wash buffer (20 mM Na-P pH 7.5, 300 mM NaCl, 15 mM imidazole, 5 % glycerol), for 1 hour on a rotating wheel. After binding, the medium was transferred into an open gravity flow column and the flow-through was discarded. Residual contaminations were removed with 15-bed volumes of wash buffer and the protein was eluted with 5-bed volumes of elution buffer (20 mM Na-P pH 7.5, 150 mM NaCl, 400 mM imidazole, 5 % glycerol). Addition of TEV protease to the eluted protein and dialysis overnight at room temperature (RT) against 1 L of dialysis buffer (20 mM Na-P pH 7.5, 300 mM NaCl, 5 % glycerol), allowed the removal of the His_6-tag. A second IMAC step separated the TEV protease and the cleaved protein, which eluted in the flow-through. The cleaved protein was concentrated to ~ 5 mL, using a 5 kDa molecular weight cut-off (MWCO) concentrator, and applied to a Superdex 75 16/600 gel filtration column (GE Healthcare), pre-equilibrated with 1× phosphate-buffered saline (PBS). Finally, the purified protein was concentrated to a concentration of 1 – 5 mg/mL, flash-frozen in liquid nitrogen and stored at -80 °C. Yields were typically in the range of 10 mg of protein per liter of culture.

Cloning, Expression and Purification of Membrane Proteins

All purifications steps were performed at 4 °C using an Äkta pure chromatography system and all buffers contained 0.03 % DDM Anagrade unless otherwise stated.

The mechanosensitive channel T2 from *Thermoplasma volcanium* was cloned, expressed, and purified, as described previously (Löw et al., 2013a) with minor modifications. In short, the gene coding for the T2 channel (accession no. TVN0821) was cloned into the expression vector pNIC28-Bsa4, which allows the expression of T2 with an N-terminal His_6-tag and a TEV protease cleavage site for tag removal. The channel was overproduced in *E. coli* C41(DE3) cells and pellets were flash-frozen in liquid nitrogen and stored at -80 °C prior to purification. In addition, the sodium phosphate buffer was replaced with 20 mM Tris buffer pH 7.5 throughout the purification, and the Ni Sepharose™ 6 Fast Flow resin was replaced with a pre-packed HisTrap HP 5 mL column (GE healthcare). The protein was eluted from the first IMAC column with a linear gradient of imidazole from 30 to 500 mM for 20 min at 1 mL/min.

The peptide transporters $PepT_{St}$ from *Streptococcus thermophilus* and $PepT_{So2}$ from *Shewanella oneidensis* were cloned, expressed, and purified as reported previously (Löw et al., 2013b; Guettou et al., 2013).

The gene coding the proton-dependent oligopeptide transporter DtpA from *E. coli* (UniProt accession number P77304) was cloned into the ptH27 expression vector (Hammarström et al., 2006). The final construct contained an N-terminal His_6-tag, a Tobacco etch virus protease (TEV) cleavage site, followed by the DtpA open reading frame and a second C-terminal His_6-tag. The protein was overproduced in *E. coli* C41(DE3) cells grown at 37 °C in TB medium supplemented with 100 μg/mL ampicillin and induced for 20 hours at 18 °C with 0.2 mM IPTG after reaching an OD_{600nm} of 0.7-0.9. The cells were harvested by centrifugation at 10,000 ×g for 15 min and stored at -20 °C.

Frozen cell pellet, from 1 L of culture, was resuspended in lysis buffer (5 mL of buffer per gram of cells; 20 mM sodium phosphate pH 7.5, 300 mM NaCl, 5 % glycerol, 15 mM imidazole pH 7.5, 0.5 mM tris(2-carboxyethyl)phosphine (TCEP), 1 mg/mL lysozyme, 5 U/mL DNase I, 100 × diluted EDTA-free complete protease inhibitor cocktail) and incubated under stirring for 45 min. The cells were then disrupted with an Emulsiflex C5 micro-fluidizer (3 to 4 passages at 15,000 p.s.i.). Unbroken cells were removed by centrifugation at 6,000 rpm for 15 min and total membranes were recovered from the first supernatant by ultracentrifugation at 35,000 rpm (Beckman Coulter Ti45 rotor) for 1 hour. Membranes were resuspended in solubilization buffer (20 mM sodium phosphate pH 7.5, 300 mM NaCl, 5 % glycerol, 15 mM imidazole pH 7.5, 0.5 mM TCEP, 100 × diluted EDTA-free complete protease inhibitor cocktail) supplemented with 1 % DDM Sol-Grade and incubated for 45 min under stirring. Unsolubilized material was removed by a second ultracentrifugation step at 30,000 rpm (Beckman Coulter Ti45 rotor) for 45 min. DtpA purification was carried out by a first IMAC step.

Solubilized membranes were incubated for 45 min on a rotating wheel with 2 mL of settled Ni Sepharose™ 6 Fast Flow resin (Invitrogen) pre-equilibrated with solubilization buffer. The solution was then poured into an open gravity flow column and the flow-through was collected and reapplied onto the resin. Unbound contaminants were removed by washing the resin with 2 × 12 mL of solubilization buffer and 2 × 12 mL of the same buffer supplemented with 40 mM imidazole pH 7.5. The protein was then eluted with 4-bed volumes of elution buffer (20 mM sodium phosphate pH 7.5, 150 mM NaCl, 5 % glycerol, 300 mM imidazole pH 7.5, 0.5 mM TCEP). Addition of 0.5 mL of TEV protease at 1 mg/mL to the eluted protein fractions followed by an overnight dialysis against 1 L of gel filtration buffer (20 mM sodium phosphate pH 7.5, 150 mM NaCl, 5 % glycerol, 0.5 mM TCEP) allowed the removal of the N-terminal His_6-tag. The cleaved protein was concentrated using a 50 kDa MWCO concentrator and further purified with a HiLoad Superdex 200 16/60 GL column (GE Healthcare). The DtpA-containing peak fractions were pooled, concentrated, and flash frozen in liquid nitrogen, and stored at -80 °C.

Lipid Preparation and Screening for SapNP Formation

Except for the brain total lipid extract (porcine), which was in powder form, all lipids stocks were purchased in chloroform-solubilized form. Lipid films were formed in glass tubes by evaporating the chloroform under a nitrogen stream. The lipid films were then incubated at RT for ~2 hours under stirring in 1× PBS and various concentrations of DDM. The turbid solutions were further incubated for 1 hour at 37 °C under shaking. Initial DDM concentration was 0.28 % (w/v) for a final lipid concentration of 5 mg/mL. However, in case the lipid of interest was still not fully solubilized (milky solution), the DDM concentration was increased in 0.1 % (w/v) steps with intermittent vortexing until the solution turned clear. In three cases, the addition of extra amounts of N,N-Dimethyldodecylamine N-oxide (LDAO) was necessary to fully solubilize the lipid. For a complete lipid list and a detailed description of the detergent concentrations, see Table S1.

The four homologous saposin proteins (A-D) were screened against a library composed of 17 different lipids and lipid mixtures for saposin nanoparticles formation. A range of lipid to saposin molar ratio (i.e., 0, 3, 6, 12, 25 and 50) was tested. In detail, the saposin protein and the lipid were mixed and incubated at 37 °C for 10 min. Detergent-free 1× PBS was then added and the mixtures were incubated for 10 min at 37 °C. The added volume of the detergent-free buffer was calculated for each saposin/lipid pair in such way that the final DDM concentration was below 0.01 % (w/v). The samples were centrifuged at 13,000 rpm for 20 min using a bench-top centrifuge prior to SEC analysis. Typically, we used 40 μl of saposin at 1 mg/mL for each ratio. For lipid mixtures, an average molecular weight of 780 Da was used to estimate the molar ratio.

Apparent Molecular Weight of SapNPs

The apparent molecular weights of different SapNPs were calculated based on the SEC retention volumes. For that, we injected the same volume (20 μL) of different SapA/lipid systems prepared at different ratios on the same SD200 5/150 home-packed column, which was pre-calibrated with 20 μL of the gel filtration standard kit from Bio-Rad.

SapNPs Physical Stability Over Time

To evaluate the stability over time of the SapNPs, equivolume fractions of SapA/SoyPI and SapA/POPG, prepared with a lipid to SapA molar ratio of 12 and stored at 10 °C, were injected on the gel filtration column after incubation at selected time points and the elution profiles were monitored by fluorescence.

Triple-Detection SEC Conjugate Analyses

The molecular weight of the T2 channel and the number of bound DDM molecules were calculated using triple-detection SEC (Gimpl et al., 2016). 60 μL of T2 in 20 mM Tris pH 7,5, 150 mM NaCl, 5 % glycerol, and 0.03 % DDM (before concentration) at 5 mg/mL were injected onto a home-packed SD200 10/300 column pre-equilibrated with the same buffer at a flow rate of 0.5 mL/min. The column was mounted on the 1260 Infinity Bio-inert high-performance liquid chromatography system (Agilent Technologies), and the elution profiles were successively monitored by UV at 280 nm, by refractive index, and by multiple angle light scattering using an in-line refractometer (Optilab rEX, Wyatt Technology), and a mini DAWN™ TREOS system equipped with a quasi-elastic light scattering module (Wyatt Technology). The refractive index increment (d*n*/d*c*) of the T2 channel was estimated using the SEDFIT software (http://www.analyticalultracentrifugation.com/default.htm) and we used a d*n*/d*c* of 0.1435 mL/g for DDM. Data were analyzed using the ASTRA 7.0 software.

Reconstitution of Membrane Proteins into SapNPs

Only SapA and detergent-free PBS were used for the reconstitution of the MPs unless otherwise stated. To reconstitute the T2 channel, four different lipids (SoyPI, POPA, POPS, and DOPC) were used. $PepT_{So2}$, $PepT_{St}$, and DtpA were reconstituted in BL, POPA, and POPS. For each of the above-mentioned MPs, we tested a combination of MP:SapA:lipid molar ratios (Table S2). Typically, 3 to 5 μL of MP at a concentration of 5 to 10 mg/mL were used for each ratio tested. The samples were prepared as follows: *i)* DDM-solubilized lipids were incubated for ~10 min at 37 °C, *ii)* the purified MP of interest was mixed with the lipids and incubated for 10-20 min at RT, *iii)* SapA was added and the mixture was incubated for 30 min at RT, and *iv)* the samples were diluted with detergent-free buffer and incubated for 10 min at RT. This last step reduced the DDM concentration to ~ the critical micelle concentration. Subsequently, the

samples were centrifuged at 13,000 rpm for 30 min using a bench-top centrifuge. In order to completely remove the remaining DDM molecules and to separate the SapNP-reconstituted MP from the empty SapNPs and the free SapA, all the samples were subjected to a SEC step.

Reconstitution of the Mechanosensitive Channel into SapNPs Using Bio-Beads and Dialysis

In the perspective of simplifying the reconstitution process and to avoid dilution for upscale preparations, the T2 channel was reconstituted in SapA/POPA using either Bio-Beads or dialysis instead of dilution for DDM removal. We used a T2:SapA:POPA molar ratio of 1:15:40 and followed the same protocol described above except that after the third step the T2 channel was treated as follow: *1)* Bio-beads were added to a final amount equivalent to 10 times the DDM mass present in the sample ($\sim$ 45 mg/ml) and incubated overnight at RT on a rotating wheel, or *2)* dialyzed against 1 L of detergent-free PBS buffer for 48 hours at RT using a 3.5 kDa MWCO dialysis cassette.

Analytical Gel Filtration Setup and Data Analysis

All small-scale screenings, to monitor the formation of SapNPs and to follow the reconstitution of MPs, were evaluated using analytical SEC. A Superdex 200 (SD200) 5/150 home-packed column (GE Healthcare) equilibrated with detergent-free 1× PBS was used on the 1260 Infinity Bio-inert high-performance liquid chromatography system (Agilent Technologies). The system operates at 10 °C and is equipped with an auto-sampler allowing the consecutive auto-injection of more than 200 samples. In addition, an inline dual detection system permits the parallel detection of the UV absorption at multiple wavelengths and fluorescence ($\lambda_{excitation}$ = 280 nm and $\lambda_{emission}$ = 350 nm). Typically 50 µl of sample were injected on the gel filtration column and analyzed in duplicates.

In order to estimate the efficiency of a saposin/lipid pair to form SapNPs, we calculated the percentage of the saposin converted to SapNPs using the formula [(1 – (the area under the free saposin peak at a given lipid to saposin molar ratio ÷ the area under the free saposin peak at a lipid to saposin molar ratio of 0)) * 100]. From these calculations, two heat maps were generated for lipid:saposin molar ratios of 6 and 12 using matplotlib (Hunter, 2007).

Protein Thermal Unfolding and Ligand Binding

The stability of the different DDM-solubilized or SapNP-reconstituted proteins was followed using a nanoDSF (Prometheus NT.48 form NanoTemper Technologies, GmbH). The dye-free proteins were excited at 280 nm and their intrinsic fluorescence at 350 and 330 nm were measured as a function of temperature to monitor the fluorescence changes upon heat unfolding. 10 µL of protein solution at a concentration of 0.5 mg/mL were loaded in a glass capillary, and the unfolding was measured in duplicates at a heating rate of 1 °C/min between 20 and 95 °C. The first derivative of the unfolding curves (350/330 nm ratio) was used to determine the transition midpoint (T_m).

$PepT_{St}$ and $PepT_{So2}$ di and tripeptide binding in solution was measured by MST with the Monolith NT.LabelFree (NanoTemper technologies). The proteins were kept at a constant concentration of ~125 nM and were combined with a series of 16 different peptide concentrations. For DDM- and SapNP-solubilized proteins, the ligands were diluted in DDM buffer (20 mM Tris pH 7.5, 150 mM NaCl, 5 % glycerol, and 0.03 % DDM) and in 1 × PBS, respectively. The thermophoretic movement of the proteins, alone and in complex with different peptides, was monitored and quantified using time-dependent intrinsic tryptophan fluorescence. The samples were loaded in standard glass capillaries. Data were acquired at 20 °C, the LED power was set to 20 %, and the MST power to 20 % and 40 %. The dissociation constant (K_D) values were derived from the nonlinear fitting of the normalized fluorescence signal at different ligand concentrations using the NT analysis software. Empty SapNPs were used as control measurements.

For the T2/N21 complex formation, the mechanosensitive channel was reconstituted alone (as described above) in SapA/POPS using a T2:SapA:POPS molar ratio of 1:15:40, and subjected to a SEC step. The fractions containing the reconstituted protein were pooled and the nanobody was added on top to a T2:N21 molar ratio of 1:1.2. The mixture was incubated for 2 hours on ice and the putative complex was then subjected to a second SEC step. All the samples were subsequently analyzed with denaturing Sodium dodecyl sulfate polyacrylamide gel electrophoresis.

SAXS Measurements

SAXS data were recorded on the EMBL P12 beamline (Blanchet et al., 2015) (DESY, Hamburg), at a wavelength of 1.24 Å using a PILATUS 2M pixel detector (DECTRIS, Switzerland) positioned at a distance of 3.1 m from the sample. The useful range of momentum transfer was $0.01 \leq s \geq 0.46$ Å-1 ($s = 4\pi \sin\theta / \lambda$, where 2θ is the scattering angle, and λ is the X-ray wavelength). Beamline configuration, data collection, and SAXS-derived parameters are detailed in Tables S3 and S4. Data were collected for three saposin/lipid systems (SapA/DOPC, SapA/SoyPI, and SapA/POPG) and two MPs (T2 and DtpA), using a standard 'batch' mode and/or the inline SEC-SAXS setup available on P12. The MPs were either solubilized in 0.03 % DDM or encapsulated in SapA/POPS NPs. For batch experiments, the samples were loaded using an automated sample changer. Dilution series were measured in corresponding buffers while flowing through a temperature controlled capillary at 20 °C and 20 frames, of 0.05 s exposure time, were collected. For the inline SEC-SAXS measurements, 60 µL were injected onto a home-packed SD200 10/300 column (GE Healthcare) pre-equilibrated in corresponding buffers and at a flow rate of 0.5 mL/min. A total of 3600 × 1 second SAXS data frames were recorded during elution. SapNPs were prepared as described above using a lipid to SapA molar ratio of 12. DDM-solubilized MPs were in 20 mM Tris pH 7.5, 150 mM NaCl, 5 % glycerol, and 0.03 % DDM (for the T2 channel) and in 20 mM sodium phosphate pH 7.5, 150 mM NaCl, 5 % glycerol, 0.5 mM TCEP, and 0.03 % DDM (for DtpA). T2 and DtpA reconstituted in SapA/POPS were prepared as described above

using a MP:SapA:POPS molar ratio of 1:15:40 and 1:20:35, respectively. Empty SapNPs and reconstituted proteins were in 1× PBS supplemented with 5 % glycerol. All samples were purified using SEC prior to data collection.

Based on comparison of successive frames, no detectable radiation damage was observed. Data from the detector were normalized to the transmitted beam intensity, averaged, placed on absolute scale relative to water and the scattering of buffer solutions was subtracted. All data were processed using PRIMUSqt from the ATSAS software package (Franke et al., 2017). SEC-SAXS data were analyzed using CHROMIXS (Franke et al., 2017).

The forward intensity *I(0)* and radius of gyration, R_g were estimated from Guinier analysis, assuming that at very small angles (s $\leq$ 1.3/R_g) the intensity is represented as $I(s)=I(0)\exp(-(sR_g)2/3)$). These parameters were also calculated from the full scattering curves using the indirect Fourier transform method implemented in the program GNOM, along with the pair distance distribution function *p(r)* and the maximum particle dimensions D_{max}.

Low-resolution *ab initio* model reconstruction from experimental SAXS data was conducted using the program DAMMIF (Franke and Svergun, 2009), where a densely packed interconnected configuration of beads that best fits the experimental data $I_{exp}(s)$ was used to search for the macromolecular shape by minimizing the discrepancy:

$$\chi 2 = \frac{1}{N-1}\sum_j \left[\frac{I_{exp}(S_j) - cI_{calc}(S_j)}{\sigma(S_j)}\right]^2$$

where N is the number of experimental points, c is a scaling factor and $I_{calc}(S_j)$ and $\sigma(S_j)$ are the calculated intensity and the experimental error at the momentum transfer S_j, respectively. For each data set, 10 independent reconstructions were made to verify the stability of the solution and the program suite DAMAVER (Volkov and Svergun, 2003) used to calculate the average and representative models. Following the initial P1 reconstructions a subsequent round was conducted for T2 using P7 symmetry. SAXS data has been deposited at the SASBDB (www.sasbdb.org). See Data Availability for details.

Superposition of *ab initio* models with the crystallographic model of the homologous T2 mechanosensitive channel protein from *T. tengcongensis* (PDB ID: 3T9N) was made using the program SUPALM (Konarev et al., 2016). SapA protein monomers (PDB ID. 4DDJ) were placed manually into the *ab initio* shape volume around the best fitting T2 superposition.

DATA AND SOFTWARE AVAILABILITY

Software Availability

SAXS data were processed and analyzed using the ATSAS suite. Visualization and image preparation was performed with Chimera, Excel, and Adobe Illustrator.

Data Availability

The SAXS data have been deposited at the SASBDB (www.sasbdb.org) and have been assigned the following accession codes: SASDC27 (SapA/DOPC), SASDC37 (SapA/SoyPI), SASDC47 (SapA/ POPG), SASDCX6 (T2 in SapA/POPS), SASDCY6 (T2 in DDM), and SASDCZ6 (DtpA in SapA/POPS).

Structure, Volume *26*

Supplemental Information

Saposin Lipid Nanoparticles: A Highly Versatile and Modular Tool for Membrane Protein Research

Ali Flayhan, Haydyn D.T. Mertens, Yonca Ural-Blimke, Maria Martinez Molledo, Dmitri I. Svergun, and Christian Löw

Saposin lipid nanoparticles: a highly versatile and modular tool for membrane protein research

Ali Flayhan[1], Haydyn D. T. Mertens[2], Yonca Ural-Blimke[1], Maria Martinez Molledo[1], Dmitri I. Svergun[2] and Christian Löw*[1, 3, 4].

[1] Centre for Structural Systems Biology (CSSB), DESY and European Molecular Biology Laboratory Hamburg, Notkestrasse 85, D-22607 Hamburg, Germany.

[2] European Molecular Biology Laboratory Hamburg, Notkestrasse 85, D-22607 Hamburg, Germany.

[3] Department of Medical Biochemistry and Biophysics, Karolinska Institutet, Scheeles väg 2, SE-17177 Stockholm, Sweden.

[4]Lead contact

* Correspondence: christian.loew@embl-hamburg.de

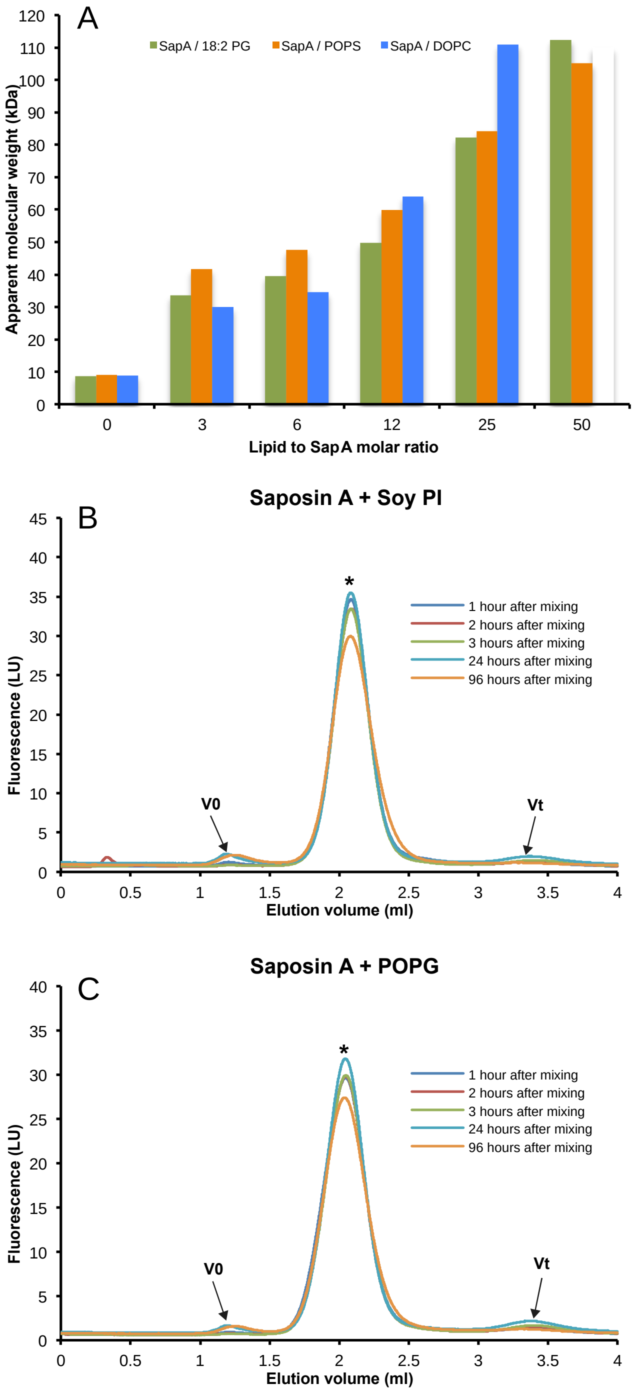

Figure S1. Related to Figure 1. (A) Positive correlation between the molecular weight of the nanoparticles and the lipid to saposin molar ratio. The lipid to SapA molar ratios are indicated on the X-axis and their corresponding apparent molecular weights, estimated by size-exclusion chromatography, on the Y-axis. The different SapA/lipid systems are color-coded as indicated in the figure. (B - C) Stability of SapNPs over time. Size-exclusion chromatography of SapA after incubation with detergent-solubilized SoyPI (B) and POPG (C), respectively. SapNPs (*) have been injected on the gel filtration column after different time points as indicated in the figure. The black arrows represent the void volume (V0) and the total volume (Vt) of the column.

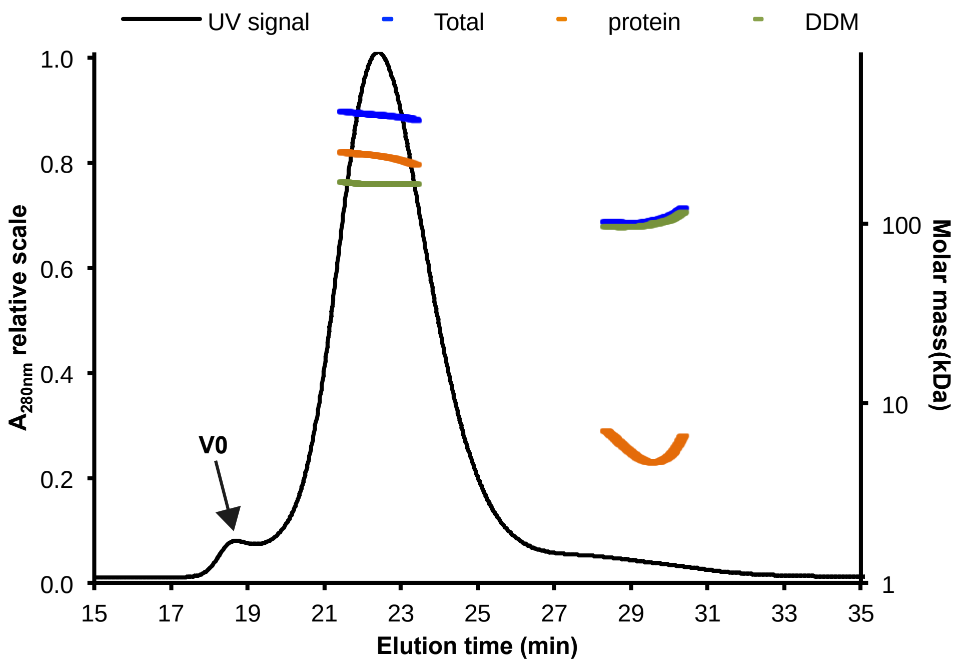

Figure S2. Related to Figure 3 and 6. Triple detection size-exclusion chromatography conjugate analysis of the T2 channel in DDM. The UV absorbance at 280 nm and the derived molar masses (logarithmic scale) of T2 (orange), DDM (green) and T2/DDM protein/detergent complex (blue) are plotted with respect to the elution time. The black arrow represents the void volume (V0) of the column. 60 µL of T2 at 5 mg/mL were injected onto a home-packed SD200 10/300 column pre-equilibrated with 20 mM Tris pH 7.5, 150 mM NaCl, 5 % glycerol, and 0.03 % DDM at a flow rate of 0.5 mL/min. Note that the second peak contains only the excess of DDM micelles.

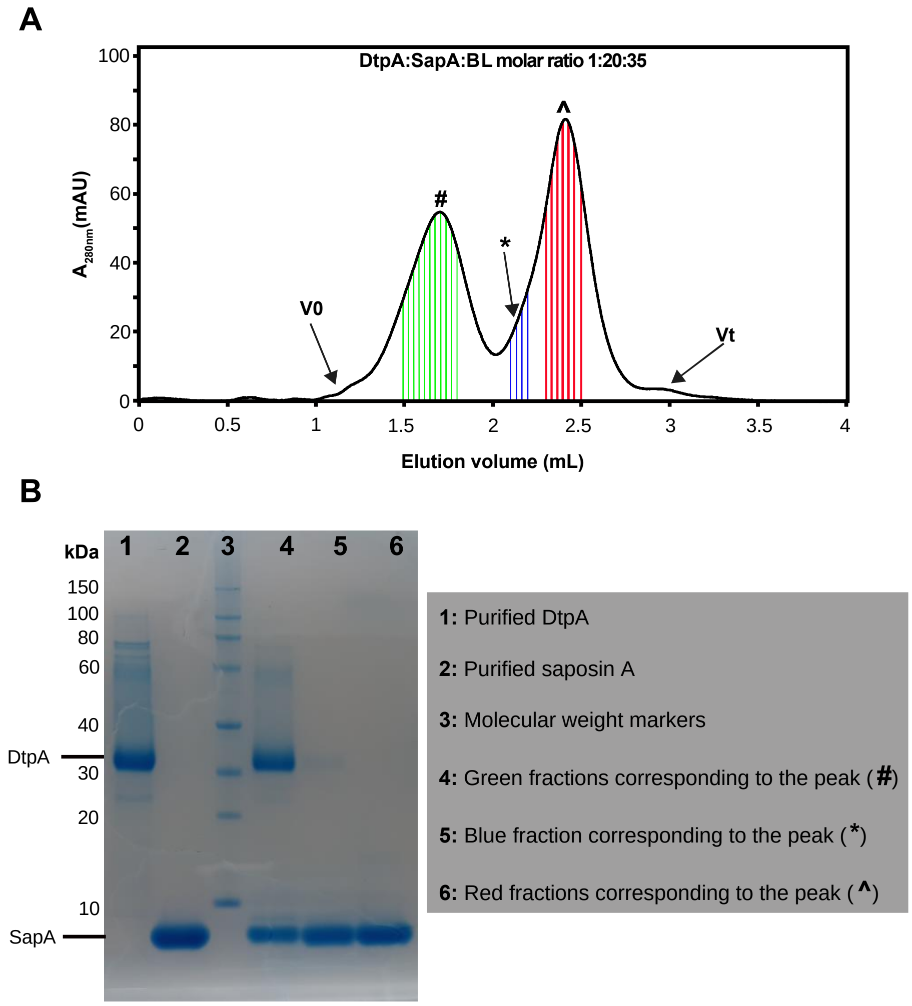

Figure S3. Related to Figure 3. (A) Gel filtration elution profile of DtpA reconstituted in SapA/BL nanopartilces. The DtpA:SapA:lipid molar ratio is 1:20:35. The free SapA peak is marked with (^), the empty nanoparticles with (*), and the reconstituted DtpA with (#). The black arrows represent the void (V0) and the total (Vt) volumes of the column used. (B) SDS-PAGE analysis of the chromatographic peaks. As seen in lanes 4 to 6, the first peak (green in panel A), assigned for the reconstituted protein, contains both DtpA and saposin A, the second and third peaks (blue and red in first panel), assigned for empty nanoparticles and free SapA, respectively, contain only SapA.

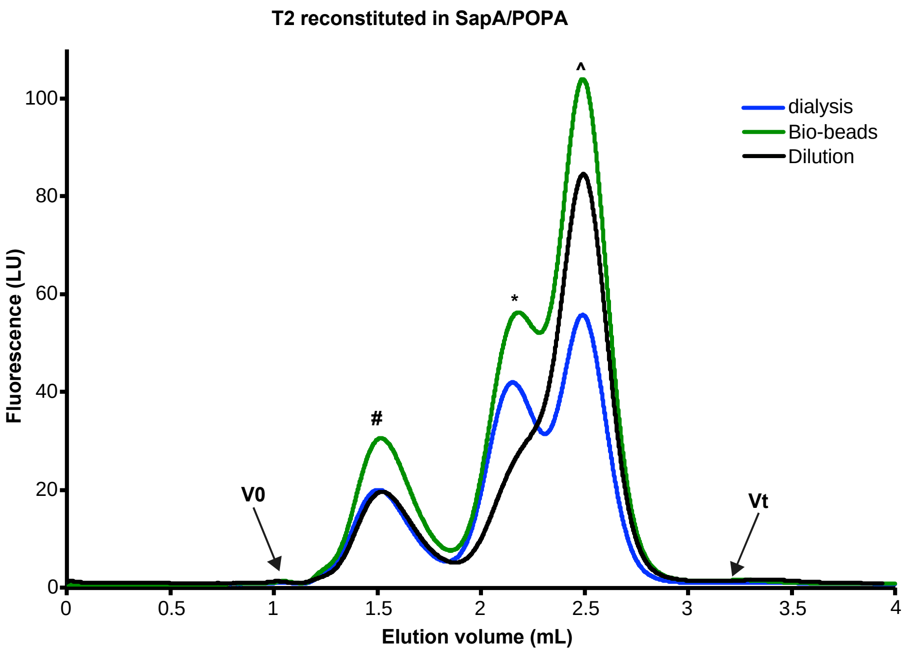

Figure S4. Related to Figure 3 and 4. Detergent removal after reconstitution. The gel filtration profiles of the mechanosensitive T2 channel after reconstitution in SapA/POPA are shown. DDM removal was carried out by dilution, dialysis, and bio-beads as indicated in the figure. The free SapA peak is marked with (^), the empty nanoparticles with (*), and the reconstituted T2 with (#). The black arrows represent the void volume (V0) and the total volume (Vt) of the column.

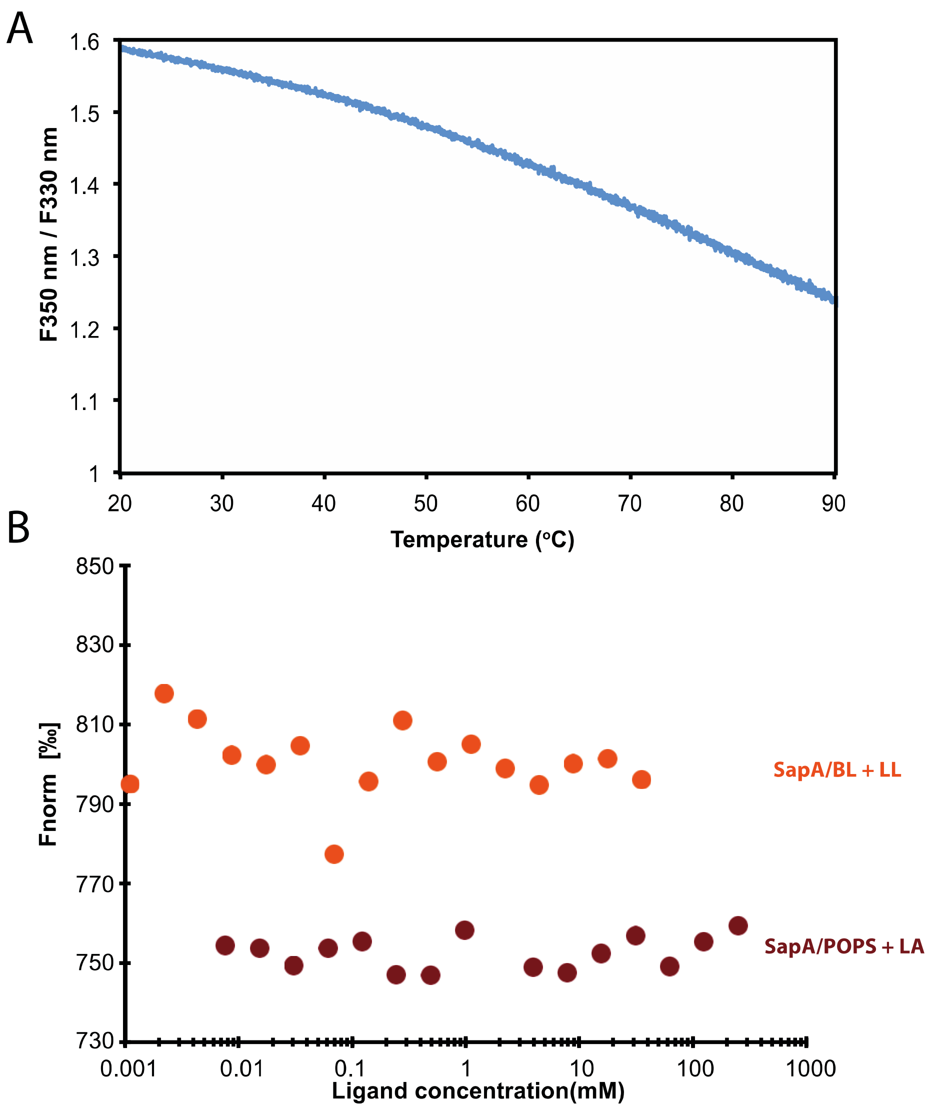

Figure S5. Related to Figure 4. (A) The experimental melting curve of purified SapA obtained with dye-free nanoDSF. Note that, the fluorescence signal of saposin A is temperature dependent as expected but does not show any obvious unfolding transition. (B) MST measurements of the binding of Leu-Leu and Leu-Ala dipeptides to SapA/BL and SapA/POPS empty nanoparticles, respectively. Unlike for the reconstituted proteins, no peptide binding could be observed with empty nanoparticles.

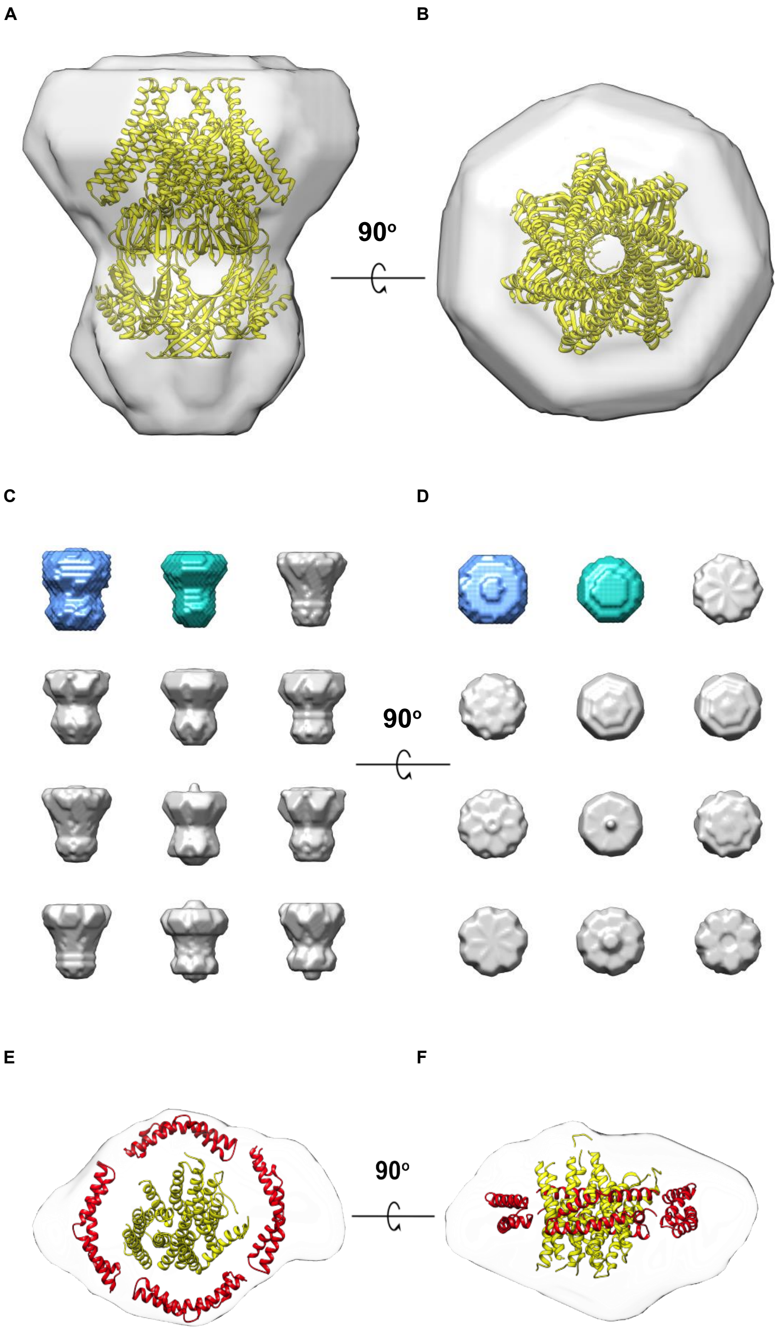

Figure S6. Related to Figure 6. (A – B) The *ab initio* model generated for the T2 channel in DDM with a homologous structure (PDB ID: 3T9N) fitted in the shape volume. Ten independent reconstructions were generated using DAMMIF (C and D, in gray) and the program DAMMAVER was used to generate the average representative models (C and D, average model blue and volume filtered cyan). *The* D_{max} of the particles is ~ 165 Å. (E – F) The best representative *ab initio* model generated for DtpA in SapA:POPS. Four SapA molecules (PDB ID: 4DDJ) and one homologous transporter molecule (PDB ID: 4Q65) were docked manually into the volume.

Table S1: A list of the lipids used for SapNPs formation. **Related to the STAR Methods section.**

**Lipid name**	**Abbreviation**	**Percentage of DDM (w/v) used to solubilize the lipid at 5 mg/ml**
L-α-phosphatidylinositol (Soy)	Soy-PI	0.5
1-palmitoyl-2-oleoyl-*sn*-glycero-3-phospho-L-serine	POPS	0.8
1-palmitoyl-2-oleoyl-*sn*-glycero-3-phospho-(1'-*rac*-glycerol)	POPG	0.6
1-palmitoyl-2-oleoyl-*sn*-glycero-3-phosphoethanolamine	POPE	1
1-palmitoyl-2-oleoyl-*sn*-glycero-3-phosphate	POPA	1
1,2-Dioleoyl-sn-glycero-3-phosphocholine	DOPC	1
1,2-dilinoleoyl-*sn*-glycero-3-phospho-(1'-*rac*-glycerol)	18:2 PG	0.8
1,2-dilinoleoyl-*sn*-glycero-3-phosphoethanolamine	18:2 PE	2.2
1,2-dilinoleoyl-*sn*-glycero-3-phosphate	18:2 PA	1.2
1,2-dilinoleoyl-*sn*-glycero-3-phosphocholine	18:2 (cis) PC	1.2
1-myristoyl-2-palmitoyl-*sn*-glycero-3-phosphocholine	MPPC	0.6
1,2-dibehenoyl-*sn*-glycero-3-phosphocholine	22:0 PC	n/a
1,2-dilauroyl-*sn*-glycero-3-phospho-(1'-*rac*-glycerol)	DLPG	0.6
D-lactosyl-ß-1,1'-N-stearoyl-D-*erythro*-sphingosine	C18 ß-D-Lactosyl Ceramide	2 and 2.5 % LDAO
D-galactosyl-ß-1,1'-N-stearoyl-D-*erythro*-sphingosine	C18 ß-D-Galactosyl Ceramide	2 and 2.5 % LDAO
1',3'-bis[1,2-dioleoyl-*sn*-glycero-3-phospho]-*sn*-glycerol	18:1 Cardiolipin	2.5 and 0.5 % LDAO
E. coli polar lipid extract	n/a	0.7
Brain total lipid extract (porcine)	BL	0.28

Table S2: Screening of different molar ratios to reconstitute membrane proteins into SapNPs**. Related to Figure 3.** Abbreviations: MP: membrane protein.

**MP**	**Lipid**	**Saposin**	**Molar ratios**	
			**Lipid to MP**	**Saposin to MP**
T2	Soy PI	SapA	40	10
				15
				20
				25
T2	POPA	SapA	40	10
				15
				20
				25
T2	POPS	SapA	40	10
				15
				20
				25
T2	DOPC	SapA	40	10
				15
				20
				25
$PepT_{SO2}$ and $PepT_{St}$	BL	SapA	35	10
				15
			70	10
				15
$PepT_{SO2}$ and $PepT_{St}$	POPA	SapA	35	10
				15
			70	10
				15
$PepT_{SO2}$ and $PepT_{St}$	POPS	SapA	35	10
				15
			70	10
				15
DtpA	BL	SapA	35	10
				15
				20
			70	10
				15
				20
DtpA	POPA	SapA	35	10
				15
				20
			70	10
				15
				20
DtpA	POPS	SapA	35	10
				15
				20
			70	10
				15
				20

Table S3: SAXS data collection and derived parameters for empty SapNPs. **Related to Figure 2.** Abbreviations: R_g: radius of gyration; D_{max}: maximal particle dimension.

	**SapA/DOPC**	**SapA/SoyPI**	**SapA/POPG**
**Data collection parameters**			
Instrument	EMBL P12 beam line (PETRA-III, DESY, Hamburg)		
Beam geometry	0.2 x 0.12 mm^2		
Wavelength (Å)	1.24		
s range ($Å^{-1}$)[a]	0.01-0.46		
Collection mode	Batch		SEC-SAXS
Exposure time (s)	1 (20×0.05 s)		3600 (3600 x 1 s)
OD_{280nm} (A.U.)	4.3	5.1	6.7
Temperature (K)	283		
**Structural parameters**			
I(0) (A.U.) (from *p(r)*)	39720 ± 51	42510 ± 37	823 ± 2
R_g (Å) (from *p(r)*)	39.9 ± 0.5	33.9 ± 0.2	40.4 ± 0.7
I(0) (A.U.) (from Guinier)	40271 ± 55	43388 ± 36	818 ± 3
R_g (Å) (from Guinier)	41.2± 0.1	35.9 ± 0.2	40.5 ± 0.2
D_{max} (Å)	115 ± 5	85 ± 5	105 ± 5
Porod volume estimate (x10^3 $Å^3$)	96 ± 10	68 ± 7	69 ± 7
Protein dry volume calculated from sequence ($Å^3$)[b]	11160		
**Software employed**			
Primary data reduction	RADAVER		
Data processing	PRIMUS/Qt		
Ab initio analysis	DAMMIF, DAMMIN		
Validation and averaging	DAMAVER		
Rigid-body modeling	CORAL		
Computation of model intensities	CRYSOL		
3D graphics representations	PyMOL, UCSF Chimera		

[a]Momentum transfer $s = 4\pi\sin(\theta)/\lambda$. [b]Dry volume determined using the server: http://www.molmovdb.org/cgi-bin/psv.cgi

Table S4: SAXS data collection and derived parameters for the T2 channel and DtpA in DDM or SapNPs. **Related to Figure 6.** Abbreviations: R_g: radius of gyration; D_{max}: maximal particle dimension.

	**T2 DDM**	**T2 SapA:POPS**	**DtpA SapA:POPS**
**Data collection parameters**			
Instrument	EMBL P12 beam line (PETRA-III, DESY, Hamburg)		
Beam geometry	0.2 x 0.12 mm^2		
Wavelength (Å)	1.24		
s range ($Å^{-1}$)[a]	0.01-0.46		
Collection mode	SEC-SAXS		
Exposure time (s)	3600 (3600 x 1 s)		
OD_{280nm} (A.U.)	10.4	3.6	11.8
Temperature (K)	283		
**Structural parameters**			
I(0) (A.U.) (from *p(r)*)	250 ± 1.0	2572 ± 5	3289 ± 3
R_g (Å) (from *p(r)*)	55.6 ± 0.2	53.9 ± 0.2	39.7 ± 0.4
I(0) (A.U.) (from Guinier)	251.22 ± 5	2563.72 ± 5	3312 ± 3
R_g (Å) (from Guinier)	56.5 ± 0.1	53.5 ± 0.2	40.2 ± 0.1
D_{max} (Å)	165 ± 5	180 ± 5	135 ± 5
Porod volume estimate (x10^3 $Å^3$)	896 ± 90	686 ± 70	288 ± 30
Protein dry volume calculated from sequence ($Å^3$)[b]	42042.5		65185.8
**Software employed**			
Primary data reduction	RADAVER		
Data processing	PRIMUS/Qt		
Ab initio analysis	DAMMIF, DAMMIN		
Validation and averaging	DAMAVER		
Rigid-body modeling	CORAL		
Computation of model intensities	CRYSOL		
3D graphics representations	PyMOL, UCSF Chimera		

[a]Momentum transfer $s = 4\pi\sin(\theta)/\lambda$. [b]Dry volume determined using the server: http://www.molmovdb.org/cgi-bin/psv.cgi
